# Biochemical Characterization of Lipids, Proteins, and Polysaccharides from the Marine Diatom *Phaeodactylum tricornutum* Cultivated in Pilot-Scale Photobioreactors

**DOI:** 10.3390/molecules31061017

**Published:** 2026-03-18

**Authors:** Arjun H. Banskota, Joseph P. M. Hui, Kaitlyn Blatt-Janmaat, Roumiana Stefanova, Alysson Jones, Sean M. Tibbetts, Patrick J. McGinn

**Affiliations:** Aquatic and Crop Resource Development Research Centre, National Research Council of Canada, 1411 Oxford Street, Halifax, NS B3H 3Z1, Canada; joseph.hui@nrc-cnrc.gc.ca (J.P.M.H.); kaitlyn.blatt-janmaat@nrc-cnrc.gc.ca (K.B.-J.); roumiana.stefanova@nrc-cnrc.gc.ca (R.S.); alyssson.jones@nrc-cnrc.gc.ca (A.J.); sean.tibbetts@nrc-cnrc.gc.ca (S.M.T.); patrick.mcginn@nrc-cnrc.gc.ca (P.J.M.)

**Keywords:** glycolipids, in vitro protein digestibility, lipid, lipidomic, protein, *Pheodactylum tricornutum*, polysaccharide, triacylglycerols, phospholipids

## Abstract

*Phaeodactylum tricornutum* was cultivated in a 1000 L photobioreactors using f/2 medium. The resulting algal biomass contained 24.5% lipids, 37.8% protein, 19.4% carbohydrates, and had a gross energy content of 19.8 MJ/kg. These components were sequentially extracted. The ultra-high performance liquid chromatography-high resolution mass spectrometry (UHPLC-HRMS) analysis of lipids revealed 35 triacylglycerols, a wide range of galactolipids and phospholipids including a novel sulfoquinovosyl diacylglycerol (SQDG), namely SQDG(C16:1/C24:0), characterized by mass fragmentation analysis. Additionally, three sulfoquinovosyl monoacylglycerols (SQMGs) with C14:0, C16:0, and C16:1 fatty acyl chain were detected in *P. tricornutum* for the first time. Fatty acid analysis further confirmed that *P. tricornutum* is an excellent source of ecosapentaenoic acid, which is predominantly present in triacylglycerol and glycolipid forms. CombiFlash chromatography allowed for the separation of monogalactosyldiacylglycerols, digalactosyldicylglycerols, SQDGs and phosphatidycholines, and their structure were confirmed by NMR spectral analysis. Fucoxanthin was the major carotenoid, and the study showed all essential amino acids required for humans and fish were present in it. A two-phase in vitro gastric/pancreatic digestibility assay showed high protein digestibility for both whole biomass (89%) and protein isolate (77%). Monosaccharide analysis showed that polysaccharides extracted by EtOH precipitation after alkaline extraction and by hot water extraction contained similar monomers with different relative intensities. Protein isolates and polysaccharides exhibited antioxidant properties.

## 1. Introduction

Diatoms are unicellular eukaryotic algae found both in marine and freshwater systems. These unique photosynthetic algae contribute significantly to global oxygen. *Phaeodactylum tricornutum* is a marine diatom belonging to the class Bacillariophyceae and is commonly found in coastal and estuarine environments. It also plays an important role in carbon fixation and as a primary producer which forms the foundation of the marine food web [1]. The genome of *P. tricornutum* revealed a mosaic of genes from green algae, red algae, and bacteria highlighting its complex evolutionary history via horizontal gene transfer [2]. It is considered a model diatom well examined at laboratory scales for production of high value bioactive compounds such as eicosapentaenoic acid (EPA), an essential long chain omega-3 polyunsaturated fatty acid (PUFA), chrysolaminarin a polysaccharide, and fucoxanthin a carotenoid having antioxidant, anti-inflammatory and anticancer properties [3]. It also emerged in the biotechnology field for recombinant protein expression host due to its biosynthetic capacity and high growth rates [4].

There is strong commercial interest in this diatom due to its potential applications in the cosmetic, food, feed, and aquaculture sectors [5,6]. Its ability to grow in saline water enables large-scale cultivation in seawater, avoiding the use of freshwater resources. *P. tricornutum* is a potentially sustainable source of lipids, including omega-3 fatty acids such as EPA, as well as pigments, proteins, and carbohydrates. Its high growth rate makes it a promising potential candidate for production of these high value chemicals with broad industrial applications in the food, feed, cosmetics and pharmaceutical industries. However, because of its complex chemical composition and siliceous frustule covering the cell wall, using whole *P. tricornutum* biomass directly as a source of protein or other high-value compounds may not be the most efficient approach. Consequently, characterizing the overall composition of biomass obtained from a sample of cultured biomass is essential for maximizing its utilization and promoting its sustainable use. In this study, we aimed to valorize *P. tricornutum* biomass cultivated in 1000 L pilot-scale Brite-Box™ photobioreactors [7]. In addition to comprehensive compositional analysis, we examined intact lipids, protein isolates, and polysaccharides. Intact lipid characterization by ultra-high performance liquid chromatography-high resolution mass spectrometry (UHPLC-HRMS) led us to the identification of 35 triacylglycerols (TAGs), 41 phospholipids, 19 sulfolipids and several dozen of galactolipids including monogalactosylmonoacylglycerols (MGMGs), monogalactosyldiacylglycerols (MGDGs) and digalactosyldiacylglycerols (DGDGs). A novel sulfolipid, namely sulfoquinovosyl diacylglycerol SQDG(C16:1/C24:0), was also identified, and the structure was well-characterized by mass fragmentation analysis. Moreover, three sulfoquinovosyl monoacylglycerols (SQMGs) were also detected for the first time in *P. tricornutum*. The antioxidant activity, especially oxygen radical absorption capacity (ORAC) value, was measured for both protein isolates and polysaccharides extracted from *P. tricornutum* to evaluate potential applications in animal feed and other high-value product development.

## 2. Results

### 2.1. P. tricornutum Culture

*P. tricornutum* biomass was collected in mid to late logarithmic phase by centrifugation and freeze-dried, with a yield of 0.22 ± 0.07 g/L. Freeze-dried algal biomass of *P. tricornutum* was dark green in color (Figure S1).

### 2.2. Compositional Analysis

**Figure 1:**
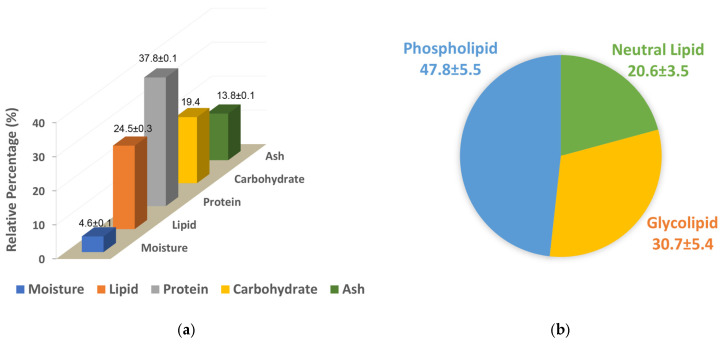
Compositional analysis of *P. tricornutum* (**a**) and the relative percentage neutral lipid, glycolipid and phospholipid fractions of total lipid separated by solid phase extraction (**b**).

The freeze-dried biomass contained 4.6% moisture. Nitrogen analysis revealed that crude protein was the predominant component, comprising 37.8% of the biomass. Lipids were the next major fraction, representing 24.5% of the total biomass. Carbohydrate content was indirectly calculated as 19.4% (Figure 1a). The ash contain of the biomass was 13.8% and the caloric density was 19.8 MJ/kg (4.7 kcal/g) gross energy.

### 2.3. Lipid Classes Separation, Fatty Acid and Carotenoid Analyses

The lipid was fractionated into neutral lipid, glycolipid and phospholipid fractions using silica gel base solid phase extraction (SPE), as described previously by Ryckebosch et al., 2012 [8], yielding 20.6, 30.7% and 47.8%, respectively (Figure 1b). Fatty acids of both lipid extract and fractions were analyzed and the results are shown in Table 1. EPA (20:5, n-3), palmitoleic acid (16:1 n-7), palmitic acid (16:0) and 6*Z*,9*Z*,12*Z*-hexadecatrienoic acid (HTA, 16:3 n-4) were the major fatty acids found in *P. tricornutum* and accounted for almost 75% of the total fatty acid detected in the lipid extract. EPA was the major fatty acid in both neutral and glycolipid fractions accounting for 37.1% and 29.3% respectively of the total fatty acid detected within the given fraction, whereas palmitoleic acid was the major fatty acid in the phospholipid fraction, accounting for 29.8% of total fatty acid followed by palmitic acid (23.4%), EPA (14.6%) and myristic acid (13.9%). Overall, PUFA accounted for >54% in neutral and glycolipid factions, whereas in the phospholipid fraction it was only 22.7%. The saturated fatty acid (SFA) and monounsaturated fatty acid (MUFA) contents in the lipid were 23.0% and 31.4%, respectively. The highest SFA content was observed in the phospholipid fraction, accounting for 41.1% of the total fatty acids within that fraction. The freeze-dried biomass contains 0.39% (*w*/*w*) of fucoxanthin. The total carotenoid content in *P. tricornutum* was 0.62% (*w*/*w*) of the freeze-dried biomass.

### 2.4. Intact Lipid Analysis

The total ion chromatograms (TICs) of neutral lipid, glycolipid and phospholipid fractions are shown in Figure S2, Figure S3 and Figure S4, respectively. The major peak detected in the neutral lipid fraction was identified as pheophytin *a*, with a pseudo-molecular ion at *m*/*z* 871.5715 [9]. Besides pheophytin, 35 triacyclglycerols (TAGs) were detected, which were eluted between 1.48 and 4.33 min in LC. The list of TAGs detected in the neutral lipid fraction of *P. tricornutum* are summarized in Table S1 with accurate mass measurement and retention time. Based on the relative intensities of individual TAG detected in the neutral lipid fraction, TAG 54:6, TAG 54:7, and TAG 54:8 were the major triacylglycerols with relative percentages of 14.1%, 13.7% and 9.7%, respectively. TAGs with acyl carbon/unsaturation 54:5, 52:4, and 48:2 each accounted for >5% of the total TAGs present in the neutral lipid fraction.

A total of one MGMG (16:2), 28 MGDGs, 10 DGDGs, two SQMGs and 16 SQDGs were identified in the glycolipid fraction (Table 2). MGDG 36:8 was the major MGDG detected in the glycolipid fraction which accounted for 24.3% of the total galactolipids followed by MGDG 36:6 (8.7%), MGDG 32:4 (8.6%), MGDG 32:2 (7.9%), MGDG 36:7 (7.5%), MGDG 40:10 (5.7%), MGDG 32:1 (5.6%) and DGDG 36:6 (5.3%). Among the sulfolipid SQDGs, 30:0, 32:1, 34:1, 30:1, and 34:2 were the major ones with relative percentages of 21.5, 18.9, 16.9, 16.7, and 13.6%, respectively, accounting for almost 87.5% of total sulfolipid detected in the glycolipid fraction. The SQMGs account for <5% of total sulfolipid based on the relative intensties of the observed molecular ions. A new SQDG, namely SQDG (16:1/24:0), was also detected in LC/MS, and structure was elucidated based on the mass spectral analysis. Two phosphatidylcholines (PCs), i.e., PC36:6 and PC36:2, were also detected in the glycolipid fraction.

In phospholipid fraction one MGMG, eight MGDGs, 10 DGDGs, 15 LPCs, 26 PCs, three SQMGs and 16 SQDGs were identified (Table 3 and Table S2). DGDG 36:6 was the major galactolipid found in phospholipid fraction with relative percentage of 37.4%. Major LPCs found in *P. tricornutum* are LPC 20:5, LPC 16:1, LPC 18:2, LPC 16:0 and LPC 18:1, having relative abundance above 3% of the total phospholipid identified. Similarly, PC 40:10, PC 36:6, PC 38:7, PC 32:2, PC 38:6, PC 32:1, and PC 34:2 have abundance higher than 5%.

### 2.5. NMR Spectral Analysis and Purification of MGDG, DGDG, SQDG and PC

**Figure 2:**
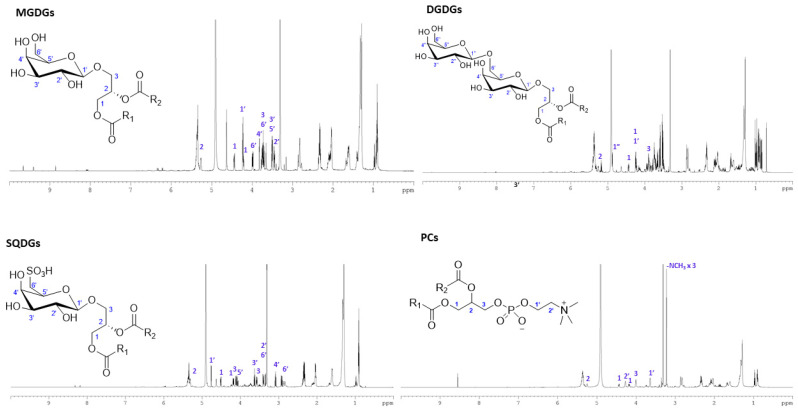
Proton NMR spectrum and assignment of diagnostic peak of sugar, glycerol or phosphocholine head groups. Clockwise from top left: MGDG-, DGDG-, PC- and SQDG-rich fractions purified from Combi Flash chromatography.

Major signals observed in proton NMR spectrum of neutral lipid fraction (Figure S5) belong to chlorophyll and fatty acids. The NMR spectrum of glycolipid and phospholipid fractions, on the other hand, showed signals belong mainly to sugars between 3.00 and 4.50 ppm, unsaturated protons between 5.20 and 5.50 ppm and methylene and methyl signals of fatty acid acyl chain between 0.80 and 3.00 ppm, clearly suggesting the presence of polar lipids such as glycolipid and phospholipids (Figures S6 and S7). Combi Flash chromatography led to purification of MGDG-, DGDG-, SQDG- and PC-rich fractions (Figure S8). The detailed procedure is described in Supplementary Document and their fractions in Table S3. The full proton NMR spectrum (0–10 ppm) of each lipid group with characteristic signals is shown in Figure 2. The structure of the sugar, glyceride or phosphocholine moities of these molecules were confirmed by analyzing 1D– (^1^H-, ^13^C-NMR) and 2D-NMR [Correlation Spectroscopy (COSY), Heteronuclear Single Quantum Coherence (HSQC) and Heteronuclear Multiple Bond Correlation (HMBC)] spectra. The key signals in ^1^H NMR spectrum, COSY and HSQC spectra of MGDGs, DGDGs, SQDGs and PCs enrich fractions shown in Figures S9–S12.

### 2.6. Protein Extraction and Characterization

Alkaline extraction followed by isoelectric precipitation yielded 40.2% protein isolate (PI) from detaffted biomass. Amino acid analysis of both freeze-dried biomass and PI suggested that glutamic acid and aspartic acid were major amino acids, each covering more than 10% of the total amino acid accounted for both whole biomass and the PI (Table 4). Relative percentage of leucine in PI was 11.3%. All essential amino acids for both human and fish were detected in *P. tricornutum* except tryptophan, which was not part of this study. The IR spectrum of the protein isolate showed peaks at 3250 cm^−1^ (O-H stretch), 2900 cm^−1^ (C-H stretch), 1640 cm^−1^ (amide I band) and 1520 cm^−1^ (amide II) (Figure S13). The sodium dodecyl sulfate-polyacrylamide gel electrophoresis (SDS-PAGE) analysis indicated that three major bands were visible in protein isolate of lower than 20 kDa in PI. Protein bands at 100 kDa and 50 kDa were visible in the SDS-PAGE analysis when whole biomass was analyzed by direct extraction of lysis buffer. The protein directly extracted from the freeze-dried algal biomass using lysis buffer showed clear bands at 100 kDa, 50 kDa, 23 kD and in addition to those of lower thah 20 kDa as observed in the PI (Figure S14).

### 2.7. Polysaccharide Extraction and Characterization

The supernatant obtained following PI extraction yielded 11.9% polysaccharides, while the final residual biomass produced 20.4% polysaccharides after hot water extraction of the defatted biomass. The IR spectra of those both polysaccharide fractions showed broad peaks at 3300 cm^−1^ belong to O-H starch. Similarly, C-H stretch at 2900 cm^−1^, 1540 cm^−1^, 1410 cm^−1^, 1050 cm^−1^ were the major peaks in both polysaccharide fractions (Figure S13). Rhamnose, fucose, ribose, xylose, mannose, galactose and glucose monomer were major sugar units of the both polysaccharide fractions and algal biomass (Figures S15–S17).

### 2.8. Two-Phase In Vitro Gastric/Pancreatic Protein Digestibility (IVPD)

Protein digestibility of both protein isolate (PI) and the freeze-dried biomass of *P. tricornutum* were tested using two-phase in vitro gastric/pancreatic protein digestibility (IVPD). Higher digestibility rate was observed for the biomass, i.e., 89.3% and the digestibility of PI was 76.7%.

### 2.9. Oxygen Radical Absorbance Capacity (ORAC) Assay

The hydrophilic ORAC values of PI and the two polysaccharide fractions were determined. PI exhibited the highest ORAC value (100.7 ± 9.7 µmole of TE/g). The polysaccharide fraction obtained by ethanol precipitation showed an ORAC value of 31.4 ± 1.2 µmole of TE/g, while the hot water extracted polysaccharide fraction exhibited an ORAC value of 53.6 ± 3.4 µmole of TE/g.

## 3. Discussion

*P. tricornutum* is a marine diatom, well known for producing high value bioactive metabolites including EPA, fucoxanthin, protein and polysaccharides [3,5,10]. Even though a number of studies have been conducted to enhance production and extraction efficiencies of target metabolites such as fucoxanthin and chrysolaminarin [11,12], none of these studies focused on separation of all possible high-value metabolites from a single sample of cultured biomass. For example, Castro-Ferreira et al., 2022 studied *P. tricornutum* extract (PTE) as a structuring agent for food applications potentially thickening or emulsifying agent containing lipid, protein and carbohydrate [10]. In the current study, *P. tricornutum* was cultivated in series of 1000 L Brite-Box™ photobioreactors and the compositional analysis revealed protein (37.8% *w*/*w*), lipid (24.5% *w*/*w*) and carbohydrate (19.4% *w*/*w*) were the major group of metabolites. The values were similar with a previous study that investigated the nutritional properties of multiple species of microalgae, including *P. tricornutum* which had protein, lipid and carbohydrate content 39.6, 18.2 and 25.2% of dry biomass, respectively [5]. High omega-3 fatty acid content, particularly high EPA level (25–28%) reported by Prestegard et al. (2016), is comparable to the values observed in the current study (25.1%), highlighting *P. tricornutum* as an excellent candidate for human and aquafeed applications [13]. The omega-3 fatty acids, namely EPA and docosahexaenoic acid (DHA), are essential components of aquafeeds and are primarily sourced from wild-caught forage fish oils, which have long since reached their plateau [14]. The aquaculture industry is continuously seeking new reliable and inexpensive sources of both proteins and oils to sustain the growing demand for nutritious seafood products and terrestrial plant-based proteins and oils derived from soy, corn, wheat and canola are already incorporated into the diets, having predominately C14–C18 fatty acids [15,16]. As a result, with the continued replacement of marine fish oil with terrestrial vegetable oils in aquafeeds, the content of highly nutritious long-chain ω-3 polyunsaturated fatty acids like EPA and DHA in farmed seafood products for the consumer has been greatly diminished in recent years [17]. Moreover, EPA has cardiovascular health benefits to humans, representing an interesting ingredient for food and nutraceutical products for human consumption [18]. Interestingly, Desbois et al. (2008) observed antibacterial activity of EPA, palmitoleic acid (C16:1 n-7) and HTA isolated from *P. tricornutum* against Gram-positive bacteria [19]. HTA further showed inhibitory activity against the Gram-negative marine pathogen *Listonella anguillarum*. In the current study we also observed that EPA, palmitoleic acid and HTA were the major fatty acids. Moreover, EPA accounted for 6.8% of the total lipid extract, which accounted for 1.7% of the freeze-dried algal biomass by weight. The relative percentage of EPA was even higher in the neutral lipid (37.1%) and glycolipid fractions (29.3%). This was further supported by observation that TAG 54:6, TAG 54:7 and TAG 54:8 were the major triacylglycerols detected in the neutral lipid fraction, possibly corresponding to EPA (20:5) containing TAGs (Table S1). Similarly, MGDG 36:8 with acyl chains of 16:3/20:5 was among the major MGDG accounting for 24.3% of the total galactolipids. Overall, the PUFA profile of *P. tricornutum* was excellent accounting for 45.6% of the total fatty acid, which was equivalent to 3.2% (*w*/*w*) of the freeze-dried algal biomass. These results strongly suggested that lipid derived from *P. tricornutum* has tremendous potential as a source of omega-3 fatty acids including EPA, as an important ingredient for human nutrition and in finfish feeds. This was further supported by the fact that *P. tricornutum* biomass has already been used as a fishmeal replacement at ratios of 3, 6 and 12% in feeds for Atlantic salmon (*Salmo salar*). The 21- and 82-day studies showed inclusion of 3 and 6% of *P. tricornutum* biomass in diets had no effect on growth, feed conversion or apparent digestibility (ADC) of protein, lipid, energy, ash and dry matter [20]. Other reports have shown that *P. tricornutum* biomass has an excellent essential amino acid (EAA) profile for fish, mink, and rodent test diets with a high EAA index (>0.9) and high nitrogen retention efficiency (39%) and moderate to high in vivo protein digestibility (69–88%). Its reported net protein utilization and biological value are somewhat lower (55–77%), which may be related to its rigid silica-based cell wall but this requires further evaluation [21]. Moreover, studies showed that feeding microalgal biomass with or without the cell wall ruptured leads to significant differences in the digestion, absorption and metabolic utilization of proteins and lipids [22,23]. It would be very interesting to investigate the effects of adding separately *P. tricornutum* extracted lipids and proteins to aquaculture feeds.

Several studies have been done on the lipid profiling in *P. tricornutum*. Lupette et al. (2019) and Li et al. (2014) characterized well the lipid in *P. tricornutum* by LC/MS identifying MGDGs, DGDGs, SQDGs, PG, TAGs, DGTSs, PIs, PCs, DGTAs and DAGs [24,25]. In a recent study, neutral lipids purified from *P. tricornutum*, predominantly composed of TAGs, were reported to exert protective effects against palmitate-induced lipid accumulation in HepG2 cells, suggesting potential applications for the treatment of non-alcoholic fatty liver disease, although intact TAG species were not analyzed [26]. The identification of 35 TAGs in the current study may support future drug development efforts for the treatment of fatty liver disease by enabling the selection and purification of major TAGs for subsequent preclinical and clinical investigations.

Glycolipids in general possess a broad spectrum of biological activities and consider as possible therapeutic application [27]. Galactolipids, especially MGDGs and DGDGs, are widely distributed in green plants and also reported to possess a broad range of biological activities [28]. Two MGDGs were reported from *P. tricornutum* as potential anticancer compounds with apoptosis-inducing activity using the patented ApopScreen cell-based screening assay [29]. Similarly, SQDGs also reported to have anti-inflammatory, anti-proliferative activity against human cancer cell lines, anti-bacterial and anti-viral properties [30,31]. These reports clearly demonstrate the pharmaceutical potential of galactolipids and sulfolipids derived from *P. tricornutum.* The identification of several MGDGs, SQMGs, and SQDGs, including the novel sulfolipid SQDG (C16:1/24:0), strongly suggests that *P. tricornutum* could serve as a valuable source of these bioactive lipids, with significant potential for application in the pharmaceutical industry.

**Figure 3:**
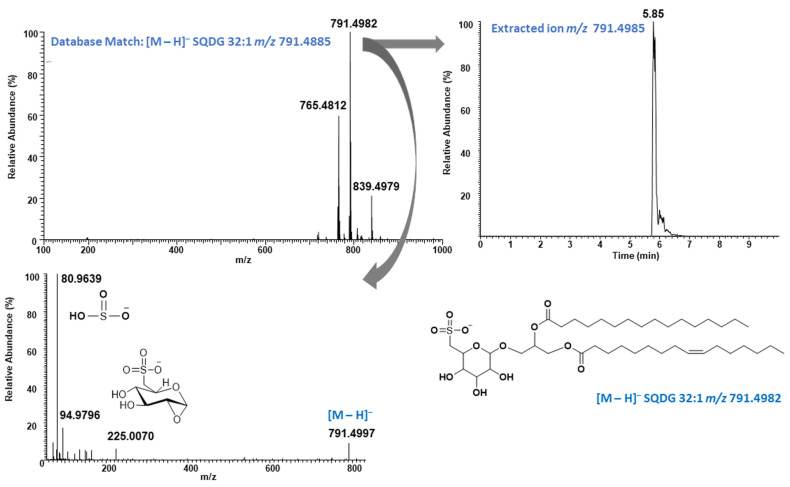
Representative SQDG 32:1 in negative mode. Clockwise from top left: ms spectrum observed at 5.85 min, extracted ion at *m*/*z* 791.4985, proposed structure and MSMS spectrum of ion at *m*/*z* 791.4982.

**Figure 4:**
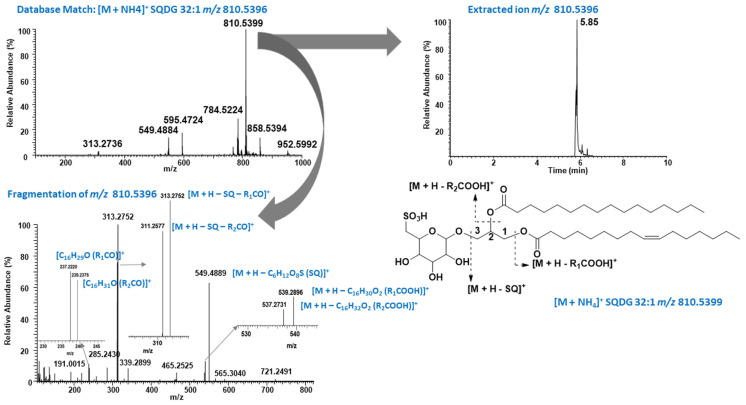
Representative SQDG 32:1 in positive mode. Clockwise from top left: ms spectrum observed at 5.85 min, extracted ion at *m*/*z* 810.5399, proposed structure and MSMS spectrum of ion at *m*/*z* 810.5399.

Negative mode of ionization is preferred for SQDG characterization and was widely reported in the literature [32,33,34,35]; very few fragmentation ions were observed for SQMG and SQDG in our study (Figure 3) besides the diagnostic ions, which limited further fatty acid assignment. In contrast, in positive ion mode the ammonium adduct ion was observed, exhibiting a diagnostic neutral loss of the sulfoquinovosyl head group (C_6_H_12_O_8_S, SQ) along with additional fragment ions (Figure 4). These fragmentation patterns are consistent with those reported by Zheng et al. (2017) in their study of yellow Sarson seeds using UHPLC coupled with triple time-of-flight mass spectrometry (UPLC–triple-TOF-MS) in ESI positive mode [36]. For example, ammonium adduct ion (M + NH_4_)^+^ at *m*/*z* 810.5399 eluted at 5.85 min matched with SQDG 32:1 in Lipid MAPS database (Figure 4). The fragment ion at *m*/*z* 549.4889 in MSMS spectrum belongs to neutral loss of SQ head group. Furthermore, the ions at *m*/*z* 539.2896 and 537.2731 were assigned to the loss of palmitic acid (R_1_COOH) and palmitoleic acid (R_2_COOH). It is well documented that the cleavage of acyl chain in *sn*-1 position is favorable compared to *sn*-2 in galactolipids such as MGDG and DGDG [37,38]. Based on this fact, regiospecific position of the fatty acid acyl group in glycerol back bone of SQDG 32:1 was proposed to be SQDG (16:1/16:0) based on the intensities of the observed ions at *m*/*z* 237.2220 and 239.2375, generated after losing one of the fatty acid acyl groups. The proposed regiospecific position of the fatty acid acyl chain was further supported by the fact that Naumann et al. (2011) well characterized SQDG 32:1 as 16:1/16:0 from *P. tricornutum* by Matrix-Assisted Laser Desorption/Ionization (MALDI) QTrap time-of-flight hybrid mass spectrometry [39]. These results clearly demonstrate that the regiospecific positions of fatty acid acyl groups in SQDG can be identified based on the relative intensities of fragment ions observed in ESI positive mode. This approach may be applied retroactively to assign the SQDG (34:2) structure as SQDG (16:0/18:2), as described by Zheng et al. 2017 (based on the MS/MS spectrum shown in the article) [36], or in future studies using ESI positive mode. The identification of the regiospecific positions of fatty acid acyl groups may play a critical role in structure activity relationship studies for drug discovery. A new SQDG, namely SQDG(16:1/24:0), was also identified in phospholipid fraction of *P. tricornutum* by mass fragmentation analysis. It was eluted at 6.37 min with molecular ion peak at *m*/*z* 903.6237 (M–H)^−^ in negative mode while at *m*/*z* 922.6667 (M + NH_4_)^+^ in positive mode (Figures S18 and S19). The *sn*-1 position was assigned for C16:1 fatty acid (palmitoleic acid) and *sn*-2 position for C24:0 fatty acid (lignoceric acid) based on the observed intensities of target fragment ions at *m*/*z* 537.2748 and 651.4100. Even though acyl position of SQMG were not assigned, it is worth to mention here that all three SQMG species with fatty acyl chain 14:0, 16:0, and 16:1 reported in *P. tricornutum* for the first time and representative MS chromatogram of SQMG 16:1 are shown in Figure S19. SQMGs have been reported to exhibit anti-tumor effects [40], suggesting that *P. tricornutum* may serve as a potential source of bioactive SQMGs for the development of cancer therapeutics.

Although LC/MS is a well-established method for characterizing complex lipid mixtures, purifying individual lipid species or specific lipid classes remains highly challenging due to their structural complexity and the presence of multiple lipid groups within crude lipid extract. Thin-layer chromatography (TLC) is a choice of technique used to separate lipid classes and identify them based on Rf values compared with standards. Yongmanitchai and Ward (1992) first reported TAG, MGDG, DGDG, SQDG, PC, and LP in a freshwater strain of *P. tricornutum* using silica cartridges [41]. Similarly, Alonso et al. (1998, 2000) further separated lipid classes in *P. tricornutum* cultured under various conditions using silica-based SPE [42,43]. TAG accounted for nearly 40% of total lipids in indoor cultures, whereas in outdoor cultures it represented only about 18%. Neutral lipids, primarily composed of TAGs, accounted for only 20.6% in the current study. Similarly, the concentrations of MAG, TAG, SQDG, MGDG, DGDG, PI, PC, PG, and PE varied with culture age and nitrogen levels [43]. Collectively, these studies demonstrated that *P. tricornutum* is an excellent source of diverse lipid classes. The separation of these lipid classes or individual lipid species is normally required for the development of high-value products, including nutraceutical, cosmeceutical, and pharmaceutical applications. The UHPLC-HRMS results (Table 2, Table 3 and Table S1) clearly demonstrated that SPE alone is insufficient for the separation of individual lipid classes. CombiFlash chromatography, which allows precise control of flow rate, solvent gradient, and collection volume, was successfully implemented to purify MGDG-, DGDG-, SQDG- and PC-enriched fractions. Structures of purified lipid fractions were confirmed by spectral analysis including 1D- and 2D-NMR spectra. These results demonstrated the CombiFlash chromatography allows for purification of these lipids groups under control conditions and may be applicable to high-value product development such as nutraceutical or pharmaceutical applications.

Pigments, particularly fucoxanthin, have been extensively studied in *P. tricornutum*. Fucoxanthin, diadinoxanthin, and β-carotene were reported by Kosakowska et al. (2004), who demonstrated that Fe(III) influences both the qualitative and quantitative pigment composition in *P. tricornutum* [44]. Similarly, Ma et al. (2011) reported a negative impact on pigment production, including chlorophyll *a* and carotenoids by chlorination and heat shocks [45]. The effects of other culture parameters on pigment production have been well documented by Celi et al. (2022) [3]. Kim et al. (2012) thoroughly characterized all-*trans*-fucoxanthin in *P. tricornutum* using spectral analysis following purification [46]. They also investigated extraction solvents and found ethanol provided the highest extraction efficiency, yielding 15.71 mg/g from a freeze-dried sample, equivalent to approximately 1.5% of the biomass. In the current study, fucoxanthin and *β*-carotene were also identified in the algal biomass by HPLC analysis (Figure S20), with total carotenoids accounting for 0.67% of the freeze-dried sample; fucoxanthin was the dominant pigment, comprising nearly 58.2% of the total carotenoids. Carotenoids are well-known antioxidants widely distributed in the plant kingdom and are responsible for the diverse colors of fruits and vegetables. They are recognized for their important role in disease prevention and the maintenance of human health [47]. Overall, *P. tricornutum* represents a good source of pigments with strong antioxidant properties and potential applications in cosmetic, nutraceutical and food industries as natural food-coloring agents.

The crude protein values determined by nitrogen count (37.8%, Figure 1a) were similar to the percentage calculated by amino acid analysis (41.9%, Table 4), which increased to 58.9% in PI [48]. The relative percentage of individual amino acids were similar both in whole biomass and PI. Glutamic acid, aspartic acid and leucine were the major amino acids found in PI which each accounted for >10% of the total amino acids, which concurs with previous report [5]. The percentage of individual amino acids, i.e., glutamic acid (8.26%), aspartic acid (8.09%) and leucine (6.62%), differed from that reported by Wang et al. (2024) indicating protein content and relative amino acid concentrations may be affected by *P. tricornatum* strain and/or the particular culture conditions used [49]. Although differences were observed in the concentrations of individual amino acids, the total essential amino acid (EAA) content of the protein isolate was 42.9%, which is comparable to the 45.44% reported by Wang et al., indicating its potential for use in food and feed applications [49]. Absence of higher molecular weight (>20 kDa) protein in PI, compared to protein-observed whole biomass (Figure S14), suggested degradation of protein during the alkaline extraction process, which was supported by the fact that Wang et al. (2024) demonstrated that pH-shift treatments altered the molecular weight distribution of *P. tricornutum* proteins and its superior foaming and emulsifying properties, highlighting the importance of extraction condition for food industry applications [49].

In vitro protein digestibility of both freeze-dried algal biomass and PI was tested using a two-phase IVPD method and found to be high for both the PI and the whole biomass (Figure 3). The digestibility of the whole biomass is consistent with the previous findings [5], which reported a similar value (89%) for whole and lipid-extracted biomass of *P. tricornutum* and is within the similar range (76–84%) reported by Wild et al. 2018 [50]. The results clearly indicate that *P. tricornutum* whole biomass may be suitable for feed applications, even without the additional protein extraction processing steps and associated costs. However, fractionating the protein isolate from the lipids and polysaccharides does enable the multiple valorizations of various components for different applications, each of which may be of higher value. It is worth mentioning here that the PI extracted from *P. tricornatum* possessed antioxidant potency with ORAC value of 100.7 μmol of TE/g. These results suggest that PI derived from *P. tricornutum* has potential applications in the food and feed industries, particularly due to its antioxidant properties, which may enhance food stability and potentially contribute to disease risk reduction. Although in vitro antioxidant assays do not directly reflect in vivo efficacy, the combination of measurable antioxidant capacity and favorable digestibility supports its promise as a functional ingredient in food and feed formulations.

Of the total, 31% of polysaccharides was recovered from the defatted biomass. Variations in peak intensities at 1580, 1430, and 1050 cm^−1^ of two polysaccharide fractions indicated differences in the chemical composition (Figure S13). Monosaccharide analysis revealed that relative higher glucose was observed in the ethanol-precipitated polysaccharide fraction, suggesting the presence of higher chrysoslaminarin, a water-soluble *β*-1,3-1,6-glucan previously reported in *P. tricornutum* and known to enhance juvenile fish health [12,51]. In addition, Costaouëc et al. (2017) reported that the cell-wall polysaccharides of *P. tricornutum*, obtained after removal of water-soluble *β*-glucans, consist of a linear poly-*α*-(1 → 3)-mannan modified with sulfate ester groups and *β*-D-glucuronic acid residues [52]. Moreover, sulfated polysaccharides with anticancer activity against HepG2 cells have also been reported from *P. tricornutum* by (Yang et al., 2019), and Guzmán et al. (2003) successfully purified polysaccharide fractions with anti-inflammatory and immunostimulatory activities using ion-exchange chromatography [53,54]. These findings suggest that *P. tricornutum* biomass contains diverse polysaccharides. In the present study, sugar analysis showed that crude polysaccharide fractions extracted by hot water or ethanol precipitation contained rhamnose, ribose, fucose, xylose, mannose, and galactose, indicating a mixture of chrysolaminarin and other polysaccharides (Figures S15 and S16). Algal whole biomass was also subjected to monomer analysis, which revealed relatively higher levels of galactose (Figure S17), possibly due to the abundance of galactolipids and sulfolipids in the biomass. Although complete structural characterization of the polysaccharides was not achieved, two distinct water-soluble polysaccharide fractions were successfully obtained. Both fractions exhibited antioxidant activity, with ORAC values of 31.4 µmole of TE/g for the ethanol precipitate and 53.6 µmole of TE/g for the hot-water extract. The ORAC values of both polysaccharide fractions and the PI were evaluated, as this assay is widely used in the food industry to express antioxidant potency. For comparison, breakfast cereals, namely corn flakes, were reported to have hydrophilic ORAC value 23.03 µmole of TE/g [55], suggesting polysaccharide from *P. tricornutum* may have potential applications as dietary fiber supplements or functional food ingredients pending further investigation.

## 4. Materials and Methods

### 4.1. General

The ^1^H NMR spectra were measured on a Bruker 700 MHz spectrometer using a 5 mm cryogenically cooled probe. IR spectrum was recorded in Nicolet 1S10 FT-IR spectrometer (Thermo Fisher Scientific, Waltham, MA, USA). HPLC analyses were carried out on an Agilent 1200 Series system (Agilent Technologies, Santa Clara, CA, USA) equipped with a diode array detector. Combi Flash chromatography was performed using Teledyne Isco (Lincoln, NE, USA). HPLC and LC/MS grade solvents were used for the extraction, solid phase extraction (SPE), Combi Flash chromatography and UHPLC-HRMS analyses.

### 4.2. Algal Culture

*P. tricornutum* strain Bohlin CCMP1327 was obtained from the National Center for Marine Algae and Microbiota (NCMA, Bigelow, ME, USA). It was grown in six, 1000 L enclosed photobioreactors over a period of several weeks in f/2 media at 22 °C, at pH 7.9 under continuous illumination. Cultures were harvested in mid to late logarithmic phase by process centrifugation (CEPA, Lahr, Germany). The accumulated biomass from 70 separate harvests was freeze-dried for 36 h at a low shelf temperature (<20 °C) in a large-capacity freeze-dryer (model 35EL, The Virtis Company, Gardiner, NY, USA). The freeze-dried algal biomass was stored at room temperature in an airtight 20 L plastic container until chemical analysis.

### 4.3. Compositional Analysis

The freeze-dried algal biomass was ground manually and filtered through laboratory sifter (Buhler AG, Switzerland) to collect flour with particle size < 0.5 mm for further analysis. Moisture, ash, crude protein, total lipid, carbohydrate, and gross energy contents were analyzed. The moisture content of freeze-dried algal biomass was measured using HES3 Moisture analyzer (Mettler Toledo, Greifensee, Switzerland); approximately 1.0 g sample was used for moisture content determination in triplicate. Ash levels were measured gravimetrically after incineration at 550 °C in a laboratory muffle furnace (model Isotemp^®^ Muffle Furnace 550-58, Thermo Fisher Scientific, Pittsburgh, PA, USA) for 18 h (AOAC 942.05, ISO 5984:2002[E]). Crude protein on algal biomass content was estimated using the average generalized N-to-P conversion factor N × 4.78 as described in the literature [48]. Total nitrogen (N) contents were determined by elemental analysis (950 °C furnace) using an N analyzer (model FP-828P, LECO Corporation, St. Joseph, MI, USA) calibrated with EDTA (LCRM^®^, Cat. #502-896), ultra-high purity oxygen as the combustion gas, and ultra-high purity helium as the carrier gas. Gross energy (GE) levels were measured using an isoperibol oxygen bomb calorimeter (model 6400, Parr Instrument Company, Moline, IL, USA) with ultra-high purity oxygen as the combustion gas, and the instrument was calibrated with benzoic acid (Parr part no. 3415). Total lipid content was determined by Folch method with modification [56]. In brief, algal sample (1.0 g) were extracted with CHCl_3_/MeOH (2:1, 10.0 mL × 3) sonicating at room temperature for 10 min in triplicate. The combined lipid extracts were dried under nitrogen and kept under vacuum overnight, weight was measured gravimetrically and total lipid content was calculated using the following formula [total lipid (%) = weight of lipid/weight of sample × 100]. The percentage of carbohydrate was calculated by using the following formula [carbohydrate (%) = 100 − moisture content − crude protein − lipid − ash content].

### 4.4. Lipid Class Separation and Fatty Acid Analysis

The lipid was fractionated into neutral lipid, glycolipid and phospholipid fractions by solid phase extraction (SPE) as described by Ryckebosch et al. 2012 [8]. Briefly, SPE column (Discovery DSC-Si Tube 3 mL 500 mg) was preconditioned by washing with 10 mL Chloroform. Approximately 100 mg of lipid dissolved in 1.0 mL chloroform was applied to the column. The column was then eluted successfully with chloroform (10 mL), acetone (10 mL) and methanol (10 mL), yielding neutral lipid, glycolipid and phospholipid, respectively. Percentage of each class of lipid was determined by their weight taken gravimetrically after drying under nitrogen followed under vacuum overnight.

Fatty acid analysis was done according to AOAC official method 991.39 (AOAC, 2000) with slight modification in triplicate [57]. Briefly, ~20 mg of lipid fractions was placed in a dry 5 mL screw-capped reaction vial and MeOH (1.0 mL) containing 0.1 mg methyl tricosanoate as an internal standard (IS). The mixture was sonicated and 1.5 N NaOH solution in MeOH (0.5 mL) was added, blanketed with nitrogen, heated for 5 min at 100 °C and cooled for 5 min. BF_3_ 14% solution in MeOH (1.0 mL, Sigma-Aldrich, St. Louis, MA, USA) was added, mixed, blanketed with nitrogen, and heated at 100 °C for 30 min. After cooling, the reaction was quenched by the addition of water (0.5 mL) and the FAME extracted with hexane (2.0 mL). Part of the hexane layer (300–600 μL) was transferred to a GC vial for analysis by GC-FID. GC-FID was carried out on an Agilent Technologies 7890A GC spectrometer (Santa Clara, CA, USA) using an Omegawax 250 fused silica capillary column (30 m × 0.25 mm × 0.25 μm film thicknesses) for fatty acid analysis. The oven temperature was held at 185 °C for 4 min, then increased to 230 °C over 11 min at 3 °C/min, then to 250 °C in 0.5 min, and kept at 250 °C for 15 min. The injector and detector temperature were set to 250 °C and 185 °C, respectively. A quantity of 1 μL of sample in hexane was injected into the column with carrier gas (helium) kept at a constant flow of 2.0 mL/min. The split ratio was 1:10. Supelco^®^ 37 component FAME mix and PUFA-3 (Supelco, Bellefonte, PA, USA) were used as fatty acid methyl ester standards. Fatty acid content in the lipid fractions was calculated by the following equation and expressed as mg/g sample. The experiment was performed in duplicate except phospholipid fraction eluted with methanol single experiment.

Fatty acid (mg/g) = (A_X_ × W_IS_ × CF_x_/A_IS_ × W_S_ × 1.04) × 1000

where A_X_ = area counts of fatty acid methyl ester; A_IS_ = area counts of internal standard (tricosylic acid methyl ester); CF_X_ = theoretical detector correlation factor is 1 except 0.99 for EPA and 0.97 for DHA; W_IS_ = weight of IS added to sample in mg; W_S_ = sample mass in mg; and 1.04 is factor necessary to express result as mg fatty acid/g sample.

### 4.5. Carotenoid Analysis

Approximately 10 mg of freeze-dried sample was extracted at room temperature by homogenizing with CHCl_3_/MeOH (1:1, 1 mL ×3) using beat beater (Bead Mill_24_, Fisher Scientific) in a 2 mL Lysing matrix Y tubes (3 × 1 min cycles). The combined extract was diluted to 10.0 mL by adding MeOH and subjected to HPLC analysis. Carotenoid analysis was performed using a YMC Carotenoid column (5 μm, 2 × 250 mm, YMC Co. Ltd., Kyoto, Japan) eluting with 50 mM NH_4_OAc in MeOH/tertiary butyl methyl ether (TBME) linear gradient 5 to 65%B in 30 min at 0.2 mL/min flow rate for 60 min. Standard curves of fucoxanthin, lutein and *β*-carotene purchased from ChromaDex (Longmont, CO, USA) were used for quantification and absorbance at 450 nm. The concentration of unknown carotenoids was calculated based on fucoxanthin standard.

### 4.6. Intact Lipid Analysis by UHPLC/HRMS

UHPLC-HRMS data were acquired on an UltiMate 3000 system coupled to a QExactive Hybrid Quadrupole-Orbitrap Mass Spectrometer (Thermo Fisher Scientific, Waltham, MA, USA) equipped with a HESI-II probe for electrospray ionization, as described previously by Banskota et al., 2022 and 2024 [58,59]. Briefly, a Thermo Hypersil Gold C8 column (100 × 2.1 mm, 1.9 μm) was used for TAG separation at 40 °C with acetonitrile/isopropyl alcohol (IPA) gradient. Initial condition was 100% acetonitrile, increased linearly to 5% IPA in 1 min, and then linearly to 70% IPA in 8 min, held for 2 min, at a flow rate of 750 µL/min. On the other hand, the glycolipids and phospholipids were separated with 10 mM ammonium acetate pH 5.0 and methanol at a flow rate of 500 µL/min, with an initial gradient of 70% methanol for the 0.25 min, increased linearly to 100% to 5 min, and held for 2.5 min. A 5 mM ammonium formate in IPA/de-ionized water/methanol 1/2/7 (*v*/*v*) solution was delivered constantly at 100 μL/min to MS going through flow splitter, via a metering pump. MS data were acquired in positive ion mode for TAG, while in both positive and negative ion modes for glycolipids and phospholipids, alternating between full MS and DataDependent MSMS scans, where the three most abundant precursor ions were subjected to MSMS using stepped normalized collision energy 20, 35 and 60%. The source parameters were set as follows: sheath gas: 15, auxiliary gas flow: 4, sweep gas: 0, spray voltage: 2.1 kV, capillary temperature: 375 °C for TAG, while 300 °C for glycolipids and phospholipids, heater temperature: 300 °C.

The identification of the individual lipids was done based on molecular ion peak matched with the LipidMAPS database [60]. Electrospray ionization (ESI) in both positive and negative modes was used for intact lipid characterization as we described in our previous study on hemp oil and microalgae *Chlorella sorokiniana* [58,59]. The ammonium adduct ions were used for the identification of TAGs. The diagnostic fragment in the positive mode at *m*/*z* 184.0734 belonging to the protonated phosphocholine head group mode was used for the identification of LPC and PC [61,62]. Similarly, SQMGs and SQDGs were identified based on the diagnostic fragment ions at *m*/*z* 225.0070 and *m*/*z* 80.9639, belonging to sulfoquinovosyl head group (C_6_H_9_O_7_S^−^) and sulphonic acid group (SO_3_H^−^), respectively in negative ESI mode (Figure 3). Ammonium adduct ion or (M + H)^+^ ions in positive mode were used to calculate relative abundance of the intact lipids except for sulfolipid where (M–H)^−^ ions were used to calculate the relative abundance (Table 2, Table 3 and Table S2).

### 4.7. Protein Isolate (PI) and Polysaccharide Extraction

The freeze-dried algal biomass (250 g) was defatted with CHCl_3_/MeOH (2:1; 1000 mL) by sonicating at room temperature 10 min followed by starring for 30 min. The resulting solution was filtered, and the residue was further extracted twice with CHCl_3_/MeOH (2:1) as described above. The combined CHCl_3_/MeOH was dried under vacuum yielding lipid (55.1 g) which was used for further lipid analysis. The defatted algal biomass was subjected to protein and polysaccharide extraction. In brief, defatted algal biomass (100 g) was mixed with 0.5 M NaOH (1.5 L) and stirred overnight at room temperature. The mixture was centrifuge at 14,000 rpm for 10 min and supernatant was collected, and pH was adjusted at 2.5 by adding dilute HCl. The resulting principate was separated by centrifuging at 4000 rpm for 20 min. The protein isolate was washed with Milli-Q water twice and freeze-dried. The protein isolate yield was 40.2 g (equivalent to 31.2% of freeze-dry algal biomass). The super supernatant collected after protein precipitation was evaporated under to reduce pressure to volume 400 mL, which was then diluted with EtOH (1600 mL) to precipitate polysaccharide. The polysaccharide was collected by centrifugation at 4000 rpm for 20 min, freeze-dried and the yield was 11.9 g (9.2% of the algal biomass). In a separate experiment (6.0 g defatted biomass) the final residual biomass collected after 0.5 M NaOH treatment was further subjected for hot water extraction at 100 °C for 1.0 h. The hot water-soluble part was separated by centrifugation at 4000 rpm for 20 min. The supernatant was freeze-dried, yielding crude polysaccharide 19.4% of the freeze-dried algal biomass.

### 4.8. Amino Acid Analysis

Freeze-dried samples or protein isolates were homogenized manually using a porcelain mortar and pestle. The algal biomass and NIST Soy flour used as standard were then hydrolyzed in triplicate using HCl (~6.0 M) at 110 °C for 24 h. The resulting extract underwent derivatization with the AccQ•Tag Ultra reagents according to the manufacturer’s protocol. Separation of the amino acid derivatives was performed by liquid chromatography on a Waters AccQ•Tag Ultra C18 column, and quantification was conducted using a UV detector monitoring at 260 nm. A gradient (99.9–40.4% A) was used for the mobile phase, where solvent A was Waters AccQ•Tag Ultra Eluent A (5% *v*/*v* in milliQ water), and solvent B was Waters AccQ•Tag Ultra Eluent B, and column temperature was maintained at 55 °C. Norvaline was used as an internal standard, and the concentration of individual amino acids are expressed in mg/g of tested sample.

### 4.9. Monosaccharide Analysis

Sugar composition of polysaccharides extracted from the algal biomass was performed by GC after hydrolysis of polysaccharides followed by acetylation as described previously [59]. In brief, polysaccharides (~20 mg) were hydrolyzed with 2M trifluoroacetic acid (TFA, 1.5 mL) at 100 °C for 1 h. The hydrolyzed product was dried under nitrogen gas and dissolved in Milli-Q water (200 μL). A drop of NH_4_OH solution was added to the solution followed by 2% sodium borohydride solution in dimethylsulfoxide (DMSO, 1.0 mL). The mixture was heated at 40 °C for 1 h and cooled down to room temperature. Glacial acetic acid (100 μL) was added followed by 1-methylimidazole (100 μL) and acetic anhydride (2.0 mL). The resulting reaction mixture was vortexed for 10 sec and kept at room temperature for 10 min. The reaction was quenched by addition of water (5.0 mL), extracted with CH_2_Cl_2_ (1.0 mL), and subjected to GC-FID analysis with an Agilent Technologies 7890A GC Spectrometer (Santa Clara, CA, USA) using Supelco SP-2340 Capillary column (60 m × 0.25 mm × 0.25 μm film thickness). The oven temperature was held at 180 °C for 6 min, then increased to 250 °C over 15 min at 2 °C/min and kept at 250 °C for 41 min. The injector and detector temperature were set to 250 °C and 180 °C, respectively. A quantity of 1 μL of sample in hexane was injected into the column with carrier gas (helium) kept at a constant flow of 2.0 mL/min. The split ratio was 1:10. Standard sugars purchased from Sigma-Aldrich (St. Louis, MA, USA), were used for identification.

### 4.10. Two-Phase In Vitro Gastric/Pancreatic Protein Digestibility (IVPD)

Protein digestibility of the *P. tricornutum* whole biomass and protein isolate was estimated using a two-phase in vitro gastric/pancreatic protein digestibility (IVPD) assay following Yegani et al. (2013) with modifications to optimize for salmonids according to Tibbetts et al. (2020) [63,64]. In brief, IVPD was measured by incubation of 250 mg of the test sample in porcine pepsin (P7000, Sigma-Aldrich) enzyme solution (25 mg/mL *w*/*v* in 0.2 N HCl at pH 1.0) for 4.5 h at 39 °C (gastric phase) with head-over-heals agitation on a Tube Rotator (model 13916-822, VWR, Mississauga, ON, Canada) equipped with Rotisserie Assembly for 50 mL tubes (model 13916-834, VWR Canada). Samples were then incubated in the same manner in porcine pancreatin containing amylase, lipase, and protease (P1750, Sigma-Aldrich) enzyme solution (100 mg mL^−1^ *w*/*v* in 0.05 M Tris, 0.0115 M CaCl_2_ buffer at pH 8.0) for 9 h (pancreatic phase) under the same incubation conditions. Assays were conducted with four analytical replicates (*n* = 4) per test sample, and each was run in parallel with procedural blanks containing no sample. The CP contents of the test samples were normalized to 100% dry matter basis by drying triplicate aliquots of each in an oven at 105 °C for 18 h. IVPD of each sample was then calculated as IVPD (%) = [(CP in test sample) − (CP in digested residue − CP in blank)]/CP in test sample × 100.

### 4.11. Oxygen Radical Absorption Capacity (ORAC) Assay

The hydrophilic ORAC assay was performed as described previously by Wu et al. (2004) with modification [55]. The assay is based on the principle that a fluorescent probe is oxidized by the addition of a free radical generator (APPH) which quenches the fluorescent probe over time. Antioxidants present in the sample block the generation of free radicals until the antioxidant activity of sample is depleted. PI and polysaccharide fractions were dissolved in 75 mM phosphate buffer (pH 7.4) 1.0 mg/mL and further diluted by phosphate buffer to have stock solution. For ORAC, 20 μL of sample (stock solution) or standard (Trolox) diluted in phosphate buffer was added to wells followed by the addition of 120 μL of fluorescein (200 nM). Plates were incubated at 37 °C for 10 min and the reaction was initiated by addition of 60 μL of 10 mM AAPH. Fluorescence decay was monitored using a fluorescence plate reader (SpectraMax Gemini XS, Molecular Devices, San Jose, CA, USA) at an excitation wavelength of 485 nm and emission wavelength of 520 nm at 37 °C. Each extract was tested in triplicate. Trolox, a water-soluble vitamin E analog, was used as the calibration standard and the results were expressed as μmole Trolox equivalents (TE)/g test sample either PI or polysaccharides.

## 5. Conclusions

*P. tricornutum* cultured in 1000 L Brite-Box™ photobioreactors using f/2 medium were harvested in mid to late logarithmic phase to study overall composition. The results clearly demonstrated that omega-3 fatty acid EPA was the major fatty acid accounting 1.7% of the freeze-dried algal biomass present in either triacylglycerol or glycolipid form. Fucoxanthin was the major carotenoid detected in *P. tricornatum.* The UHPLC/HRMS analysis led to identification of 35 triacylglycerols, glycolipids including MGMG, MGDGs, DGDGs and SQDGs and phospholipids including LPCs and PCs. A novel SQDG, namely SQDG (C16:1/C24:0), was also identified, and its structure was fully confirmed by mass fragmentation analysis. Additionally, three SQMGs with fatty acyl chain C14:0, C16:0, and C16:1 were identified in *P. tricornutum* for the first time. The regiospecific position fatty acid acyl group of SQDG was assigned using fragmentation ion in ESI positive mode. CombiFlash chromatography allowed to separate major glycolipid fractions and confirm their structure by NMR spectral analysis including 1D- (^1^H and ^13^C) and 2D-NMR (COSY, HSQC and HMBC) spectra. All essential amino acids required for both human and fish were found in *P. tricornutum*. Glutamic and aspartic acids were the major amino acids detected in the biomass and protein isolate. Protein digestibility (as estimated by two-phase IVPD) was highest for the whole biomass (89%) and marginally lower for the PI (77%). This suggests that *P. tricornutum* whole biomass may be suitable for food/feed applications, even without additional protein extraction processing steps and associated costs. However, fractionating the protein isolate from the lipids and polysaccharides could enable multiple valorizations of various components for different applications, each of which may be of higher value. The sequential extraction process provided clear separation of the lipid, protein isolate, and polysaccharide fractions, each offering potential for application in different industrial sectors. Two water-soluble polysaccharide fractions exhibited antioxidant activity, suggesting their potential use as dietary fiber supplements or functional food ingredients, subject to further investigation. The results clearly demonstrate the potential industrial applications of lipids, proteins, and polysaccharides extracted from a single biomass of *P. tricornutum*.

## Figures and Tables

**Table 1:** Fatty acid profile of lipid fractions. Results are expressed in mg/g of lipid extract or lipid fractions. The percentage of individual fatty acid in the lipid extract or lipid fractions is given in the parenthesis with respect to total fatty acid detected in the tested sample.

Fatty Acids	Total Lipid	Neutral Lipid Fr.	Glycolipid Fr.	Phospholipid Fr.
Myristic acid (C14:0)	21.0 ± (7.8)	17.6 ± 1.5 (5.1)	12.2 ± 0.3 (3.7)	41.7 ± 0.9 (13.9)
Myristoleic acid (C14:1)	-	20.3 ± 1.3 (5.9)	1.8 ± 0.1 (0.5)	-
Pentadecanoic acid (C15:0)	0.7 ± 0.1 (0.3)	0.9 ± 0.1 (0.3)	-	1.3 ± 0.0 (0.4)
*cis*-10-Pentadecenoic acid (C15:1)	-	2.5 ± 0.1 (0.7)	-	-
Palmitic acid (16:0)	35.3 ± 3.1 (13.1)	21.4 ± 1.9 (6.3)	22.5 ± 0.5 (6.7)	70.3 ± 0.6 (23.4)
Palmitoleic acid (C16:1 n-7)	73.2 ± 7.5 (27.2)	75.7 ± 6.2 (22.2)	79.5 ± 0.3 (23.8)	89.6 ± 2.3 (29.8)
C16:2 n-4	10.2 ± 1.1 (2.8)	14.4 ± 1.3 (4.2)	17.9 ± 0.1 (5.4)	6.4 ± 0.2 (2.1)
Heptadecanoic acid (C17:0)	0.1 ± 0.0 (0.0)	-	-	-
6Z,9Z,12Z-Hexadecatrienoic acid (HTA, C16:3 n-4)	25.9 ± 2.6 (9.6)	19.4 ± 1.6 (5.7)	66.6 ± 0.5 (20.0)	6.3 ± 0.2 (2.1)
C16:4 n-1	4.2 ± 0.4 (1.6)	3.8 ± 0.3 (1.1)	11.0 ± 0.1 (3.3)	-
Stearic acid (C18:0)	0.7 ± 0.1 (0.2)	2.4 ± 0.8 (0.7)	-	1.2 ± 0.3 (0.4)
Oleic acid (C18:1 n-9)	8.9 ± 0.9 (3.3)	14.0 ± 1.8 (4.1)	7.0 ± 1.8 (2.1)	15.7 ± 2.8 (5.2)
C18:1 n-7	2.2 ± 0.2 (0.8)	2.1 ± 0.2 (0.6)	1.6 ± 0.0 (0.5)	3.7 ± 0.2 (1.2)
Linoleic acid (C18:2 n-6)	4.5 ± 0.5 (1.7)	9.4 ± 1.0 (2.7)	4.0 ± 0.9 (1.2)	6.6 ± 1.5 (2.2)
C18:2 n-4	0.1 ± 0.0 (0.0)	-	-	-
*γ*-linolenic acid (C18:3 n-9)	1.0 ± 0.1 (0.4)	1.4 ± 0.5 (0.4)	1.9 ± 0.1 (0.6)	-
*α*-linolenic acid (C18:3 n-3)	0.6 ± 0.0 (0.2)	2.6 ± 0.2 (0.8)	1.8 ± 0.3 (0.6)	1.9 ± 0.5 (0.6)
C18:4 n-3	1.5 ± 0.2 (0.6)	1.7 ± 0.3 (0.5)	4.9 ± 0.0 (1.5)	-
C18:4 n-1	2.2 ± 0.2 (0.8)	-	-	-
*cis*-11-Eicosenoic acid (C20:1 n-9)	0.2 ± 0.0 (0.1)	-	-	-
*cis*-8,11,14-Eicosatrienoic acid (C20:3 n-6)	0.6 ± 0.1 (0.2)	-	-	-
*cis*-11,14-Eicosadienoic acid (C20:2 n-6)	0.2 ± 0.0 (0.1)	-	-	-
Arachidonic acid (C20:4 n-6)	0.7 ± 0.1 (0.3)	2.0 ± 1.4 (0.6)	-	-
Eicosapentaenoic acid (EPA, C20:5 n-3))	67.6 ± 7.1 (25.1)	126.6 ± 10.5 (37.1)	97.8 ± 0.4 (29.3)	44.0 ± 1.2 (14.6)
Behenic acid (C22:0)	0.5 ± 0.1 (0.1)	-	-	-
Erucic acid (C22:1 n-9)	0.2 ± 0.1 (0.1)	-	1.4 ± 0.3 (0.4)	-
C21: 5n3	0.2 ± 0.0 (0.1)	-	-	-
C22:5 n-6	0.2 ± 0.0 (0.1)	-	-	-
C22:5 n-3 DPA	0.2 ± 0.1 (0.1)	-	-	-
Lignoceric acid (C24:0)	3.7 ± 0.2 (1.4)	-	-	9.1 ± 0.1 (3.0)
Docosahexaenoic acid (DHA, C22:6 n-3))	2.3 ± 0.2 (0.8)	2.8 ± 0.3 (0.8)	1.8 ± 0.1 (0.6)	3.0 ± 0.1 (1.0)
Saturated fatty acid (SFA)	62.1 ± 5.5 (23.0)	42.3 (12.4)	34.7 (10.4)	123.7 (41.1)
Monounsaturated fatty acid (MUFA)	84.7 ± 8.7 (31.4)	114.6 (33.6)	89.9 (26.9)	109.0 (36.2)
Polyunsaturated fatty acid (PUFA)	122.9 ± 12.8 (45.6)	184.2 (54.4)	209.1 (62.7)	68.1 (22.7)
Total fatty acid	269.7 ± 26.9 (100)	341.1 (100)	333.7 (100)	300.7 (100)

- not detected; Fr. fraction.

**Table 2:** Monogalactosylmonoacylglycerol (MGMG), monogalactosyldiacylglycerols (MGDGs), digalactosyldiacylglycerols (DGDGs), sulfoquinovosyl monoacylglycerols (SQMGs) and sulfoquinovosyl diacylglycerols (SQDGs) identified in glycolipid fraction and their relative abundance.

RT (min)	Exact Mass (*m*/*z*)	Observed Mass (*m*/*z*)	Error (ppm)	Glycolipid	RI (%) GL Fraction	R1/R2
MGMG, MDDGs and DGDGs (M + NH_4_)^+^						
3.19	506.3329	506.3326	0.6	MGMG 16:2	0.4	16:02 *
5.90	738.5151	738.5152	−0.1	MGDG 32:5	4.1	16:2/16:3; 16:1/16:4
5.90	902.5835	902.5844	−1.0	DGDG 32:4	0.1	NI
5.91	952.5992	952.5994	−0.2	DGDG 36:7	2.0	16:2/20:5
5.92	788.5307	788.5306	0.1	MGDG 36:8	24.3	16:3/20:5
5.94	764.5307	764.5321	−1.8	MGDG 34:6	0.0	16:2/16:4 *
5.94	776.5307	776.5299	1.0	MGDG 34:0	0.0	16:0/18:0 *
6.00	928.5992	928.6002	−1.1	DGDG 34:5	0.1	NI
6.04	814.5464	814.5471	−0.9	MGDG 38:9	1.1	18:4/20:5
6.05	740.5307	740.5309	−0.3	MGDG 32:4	8.6	16:1/16:3; 16:2/16:2
6.05	954.6148	954.6144	0.4	DGDG 36:6	5.3	16:1/20:5
6.08	790.5464	790.5461	0.4	MGDG 36:7	7.5	16:2/20:5
6.09	904.5992	904.6000	−0.9	DGDG 32:3	0.1	16:1/16:2
6.13	742.5464	742.5463	0.1	MGDG 32:3	7.9	16:1/16:2; 16:0/16:3
6.13	766.5464	766.5463	0.1	MGDG 34:5	2.0	NI
6.13	816.5620	816.5626	−0.7	MGDG 38:8	1.1	18:3/20:5
6.13	840.5620	840.5624	−0.5	MGDG 40:10	5.7	20:5/20:5
6.13	906.6148	906.6151	−0.3	DGDG 32:2	1.3	16:1/16:1
6.17	792.5620	792.5618	0.3	MGDG 36:6	8.7	16:1/20:5
6.17	930.6148	930.6132	1.7	DGDG 34:4	0.0	NI
6.24	768.5620	768.5623	−0.4	MGDG 34:4	0.9	16:0/18:4; 16:1/18:3; 16:2/18:2; 16:3/18:1
6.33	956.6305	956.6312	−0.7	DGDG 36:5	1.7	NI
6.35	744.5620	744.5618	0.3	MGDG 32:2	7.9	16:1/16:1
6.35	818.5777	818.5773	0.5	MGDG 38:7	1.4	18:2/20:5
6.35	908.6305	908.6302	0.3	DGDG 32:1	0.5	16:0/16:1
6.35	934.6461	934.6461	0.0	DGDG 34:2	0.1	NI
6.39	746.5777	746.5776	0.1	MGDG 32:1	5.6	16:1/16:0
6.39	770.5777	770.5783	−0.8	MGDG 34:3	0.8	16:0/18:3; 16:1/12:2; 16:2/18:1
6.42	774.6090	774.6089	0.1	MGDG 34:1	0.2	16:0/18:1 *
6.51	772.5933	772.5939	−0.8	MGDG 34:2	0.5	16:1/18:1; 16:0/18:2
SQMGs and SQDGs (M–H)^−^						
2.82	553.2688	553.2690	−0.4	SQMG 16:1	0.1	16:1 *
3.39	555.2845	555.2845	0.0	SQMG 16:0	0.2	16:0 *
5.46	761.4515	761.4519	−0.5	SQDG 30:2	1.1	14:0/16:2 *
5.53	737.4515	737.4521	−0.8	SQDG 28:0	1.2	14:0/14:0
5.53	811.4672	811.4679	−0.9	SQDG 34:5	0.6	20:5/14:0
5.58	787.4672	787.4675	−0.4	SQDG 32:3	0.3	16:1/16:2 *
5.62	837.4828	837.4830	−0.2	SQDG 36:6	0.3	20:5/16:1 *
5.65	763.4672	763.4668	0.5	SQDG 30:1	16.7	14:0/16:1
5.68	789.4828	789.4828	0.0	SQDG 32:2	3.6	16:1/16:1; 16:0/16:2 (major)
5.70	839.4985	839.4981	0.4	SQDG 36:5	2.6	16:0/20:5
5.70	765.4828	765.4802	3.4	SQDG 30:0	21.5	14:0/16:0
5.85	791.4985	791.4983	0.2	SQDG 32:1	18.9	16:1/16:0
5.90	817.5141	817.5145	−0.5	SQDG 34:2	13.6	16:1/18:1; 18:2/16:0 (minor)
6.00	819.5298	819.5300	−0.2	SQDG 34.1	16.9	16:0/18:1; 16:1/18:0 *
6.37	903.6237	903.6237	0.0	SQDG 40:1	2.4	16:1/24:0

* position of fatty acid acyl chain is not assigned, NI—fatty acid acyl chain is not defined because of lack of diogenstic fragment ions; low abundance 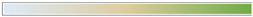 high abundance.

**Table 3:** Phosphatidylcholines (PCs) and lysophosphatidylcholines (LPCs) identified in phospholipid fraction and their relative abidance.

RT (min)	Exact Mass (*m*/*z*)	Observed Mass (*m*/*z*)	Error-ppm	Phospholipid C:DB	Relative Abundance (%)	R1/R2
2.57	490.2928	490.2929	−0.20	LPC 16:3	0.5	16:3/0:0
3.12	492.3085	492.3086	−0.20	LPC 16:2	0.8	16:2/0:0
3.35	468.3085	468.3085	0.00	LPC 14:0	0.3	14:0/0:0
3.51	542.3241	542.3244	−0.55	LPC 20:5	6.3	20:5/0:0
3.57	518.3241	518.3245	−0.77	LPC 18:3	0.7	18:3/0:0
3.61	494.3241	494.3242	−0.20	LPC 16:1	5.5	16:1/0:0
3.84	544.3398	544.3401	−0.55	LPC 20:4	1.4	20:4/0:0
3.97	568.3398	568.3400	−0.35	LPC 22:6	1.3	22:6/0:0
4.04	520.3398	520.3400	−0.38	LPC 18:2	3.5	18:2/0:0
4.26	496.3398	496.3399	−0.20	LPC 16:0	3.5	16:0/0:0
4.29	546.3554	546.3555	−0.18	LPC 20:3	1.3	20:3/0:0
4.49	522.3554	522.3556	−0.38	LPC 18:1	4.7	18:1/0:0
4.53	572.3711	572.3714	−0.52	LPC 22:4	0.1	22:4/0:0
4.72	548.3711	548.3713	−0.36	LPC 20:2	0.6	20:2/0:0
4.95	574.3867	574.3872	−0.87	LPC 22:3	0.1	22:3/0:0
5.90	822.5643	822.5647	−0.49	PC 38:6 O	1.2	NI
6.09	774.5068	774.5070	−0.26	PC 36:8	0.7	NI
6.13	726.5068	726.5075	−0.96	PC 32:4	0.9	NI
6.13	776.5225	776.5230	−0.64	PC 36:7	1.8	NI
6.13	826.5381	826.5388	−0.85	PC 40:10	5.5	20:5/20:5
6.34	704.5225	704.5242	−2.41	PC 30:1	1.3	NI
6.34	728.5225	728.5192	4.53	PC 32:3	1.4	NI
6.34	752.5225	752.5240	−1.99	PC 34:5	1.3	NI
6.34	754.5381	754.5398	−2.25	PC 34:4	1.8	NI
6.34	778.5381	778.5399	−2.31	PC 36:6	8.2	16:1/20:5 *
6.34	802.5381	802.5399	−2.24	PC 38:8	1.0	NI
6.34	804.5538	804.5554	−1.99	PC 38.7	6.9	18:2/20:5 *
6.34	828.5538	828.5551	−1.57	PC 40:9	2.2	NI
6.36	730.5381	730.5381	0.00	PC 32:2	6.4	16:1/16:1
6.36	742.5381	742.5375	0.81	PC 36:3	0.6	NI
6.36	830.5694	830.5687	0.84	PC 40:8	2.2	NI
6.45	780.5538	780.5530	1.02	PC 36:5	0.0	16:0/20:5 *
6.55	706.5381	706.5384	−0.42	PC 30:0	0.6	NI
6.55	744.5538	744.5540	−0.27	PE 36:2	0.2	NI
6.55	756.5538	756.5540	−0.26	PC 34:3	3.4	16:1/18:2 *
6.55	806.5694	806.5694	0.00	PC 38:6	6.1	18:1/20:5 *
6.58	732.5538	732.5535	0.41	PC 32:1	6.6	16:0/16:1 *
6.58	758.5694	758.5688	0.79	PC 34:2	5.7	16:1/18:1 *
6.78	784.5851	784.5854	−0.38	PC 36:3	1.3	NI
6.81	760.5851	760.5848	0.39	PC 34:1	2.3	16:0/18:1 *
7.00	786.6007	786.6010	−0.38	PC 36:2	0.8	NI

* position of fatty acid acyl chain is not assigned; NI—fatty acid acyl chain is not defined because of lack of diogenstic fragment ions; low abundance 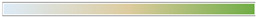 high abundance.

**Table 4:** Amino acid analysis of freeze-dried biomass and the crude protein isolate (PI). Each value represents the mean ± SD of three independent experiments. Results are expressed in mg/g of the tested sample and the relative percentage of individual amino acid with respect to total amino acid count is given in parenthesis. NIST Soy Flour standard is used as reference standard.

Amino Acids (AAs)	Freeze-Dried Biomass	Protein Isolate (PI)	NIST Soy Flour Standard
Histidine (His)	11.2 ± 3.2 (2.7)	12.4 ± 2.3 (2.1)	17.3 ± 0.4 (3.7)
Serine (Ser)	18.1 ± 0.2 (4.4)	23.2 ± 0.7 (4.0)	22.2 ± 0.5 (4.8)
Arginine (Arg)	23.6 ± 0.5 (5.7)	34.1 ± 0.8 (5.8)	35.1 ± 0.4 (7.6)
Glycine (Gly)	23.5 ± 0.4 (5.7)	37.7 ± 1.0 (6.5)	20.5 ± 0.2 (4.4)
Aspartic acid (Asp)	47.1 ± 0.8 (11.4)	68.8 ± 1.6 (11.8)	55.0 ± 1.2 (11.9)
Glutamic acid (Glu)	61.0 ± 1.3 (14.7)	73.0 ± 1.9 (12.5)	88.5 ± 1.6 (19.1)
Threonine (Thr)	20.4 ± 0.2 (4.9)	22.5 ± 0.6 (3.9)	18.4 ± 0.4 (4.0)
Alanine (Ala)	30.6 ± 0.3 (7.4)	44.4 ± 1.1 (7.6)	19.7 ± 0.3 (4.3)
Proline (Pro)	25.0 ± 0.3 (6.0)	28.4 ± 0.7 (4.9)	24.3 ± 0.3 (5.2)
Tyrosine (Tyr)	11.6 ±5.7 (2.8)	21.5 ± 0.7 (3.7)	14.9 ± 0.7 (3.2)
Lysine (Lys)	24.4 ± 2.9 (5.9)	28.0 ± 2.3 (4.8)	30.9 ± 0.5 (6.7)
Methionine (Met)	9.4 ± 2.6 (2.3)	6.1 ± 0.1 (1.0)	5.6 ± 0.0 (1.2)
XCysteine (XCys) *	4.6 ± 4.2 (1.1)	2.5 ± 0.2 (0.4)	6.1 ± 0.3 (1.3)
Valine (Val)	26.1 ± 3.5 (6.3)	23.5 ± 2.1 (4.0)	20.9 ± 0.3 (4.5)
Isoleucine (Ile)	21.2 ± 0.3 (5.1)	41.2 ± 0.9 (7.1)	23.3 ± 0.3 (5.0)
Leucine (Leu)	33.2 ± 0.4 (8.0)	66.0 ± 1.5 (11.3)	36.7 ± 0.5 (7.9)
Phenylalanine (Phe)	23.9 ± 0.4 (5.8)	50.5 ± 1.3 (8.7)	24.1 ± 0.2 (5.2)
Total AA mg/g (relative %)	414.9 (100)	583.8 (100)	463.5 (100)
EAA mg/g **	169.8 (40.9)	250.2 (42.9)	177.2 (38.2)
EAA/Total AA **	0.41	0.43	0.38

* XCys—cysteine + cystine, ** tryptophan is not included because it was not part of the study.

## Data Availability

The original contributions presented in this study are included in the article. Further inquiries can be directed to the corresponding author.

## Supplementary Materials

The following supporting information can be downloaded at https://www.mdpi.com/article/10.3390/molecules31061017/s1, Table S1: Triacylglycerols identified in neutral lipid fraction by UHPLC/HRMS analysis. [M + NH_4_]^+^ ion was used for the database search; Table S2: Glycolipids identified in phospholipid fraction and their relative intensities; Table S3: CombiFlash chromatography pooled fractions, along with their corresponding weights and recoveries; Figure S1: Freeze dry biomass of *P. tricornutum*; Figure S2: Total ion chromatogram (TIC) of neutral lipid fraction in electrospray ionization (ESI) positive mode; Figure S3: TICs of glycolipid lipid fraction in ESI positive and negative mode; Figure S4: TICs of phospholipid lipid fraction in ESI positive and negative mode. Figure S5: ^1^H NMR spectrum of neutral lipid fraction recorded in 700 MHz NMR Spectrometer; Figure S6: ^1^H NMR spectrum of glycolipid lipid fraction recorded in 700 MHz NMR Spectrometer; Figure S7: ^1^H NMR spectrum of phospholipid lipid fraction recorded in 700 MHz NMR Spectrometer; Figure S8: ConbiFlash Chromatography chromatogram of phospholipid fraction; Figure S9: Diagnostic NMR spectrum of MGDGs rich fraction ^1^H NMR, COSY and HSQC; Figure S10: Diagnostic NMR spectrum of DGDGs rich fraction ^1^H NMR, COSY and HSQC; Figure S11: Diagnostic NMR spectrum of SQDGs rich fraction ^1^H NMR, COSY and HSQC; Figure S12: Diagnostic NMR spectrum of PCs rich fraction ^1^H NMR, COSY and HSQC; Figure S13: FT-IR spectrum of protein isolate and polysaccharide fractions; Figure S14: SDS-PAGE profiles of protein isolate and protein extracted from *P. tricornutum* biomass using lysis buffer; Figure S15: Monomer analysis of polysaccharide extracted by EtOH precipitation, GC chromatogram and relative percentage of the monomer detected; Figure S16: Monomer analysis of polysaccharide extracted by hot water, GC chromatogram and relative percentage of the monomer detected; Figure S17: Monomer analysis of *P. tricornutum* whole biomass, GC chromatogram and relative percentage of the monomer detected; Figure S18: MS spectra of SQDG (*sn*-1:C16:1/*sn*-2:C24:0) eluted at 6.37 min with fragmentation in positive and negative modes; Figure S19: Total extracted ion of SQMG (C16:1) eluted at 6.37 min and MSMS fragmentation in negative and positive modes; Figure S20: HPLC chromatogram of *P. tricornutum* CHCl_3_/MeOH (1:1) extract at 450 nm and 665 nm.

## Abbreviations

The following abbreviations are used in this manuscript:

COSY	Correlattion spectroscopy
DGDG	Digalactosyl diacylglycerol
DHA	Docosahexaenoic acid
EAA	Essential amino acid
EPA	Eicosapentaenoic acid
ESI	Electrospray ionization
HMBC	Heteronuclear multiple bond correlation
HRMS	High resolution mass spectrometry
HSQC	Heteronuclear single quantum coherence
HTA	6Z,9Z,12Z-Hexadecatrienoic acid
LPC	Lysophosphatidylcholine
MGMG	Monogalactosyl monoacylglycerol
MGDG	Monogalactosyl diacylglycerol
MUFA	Monounsaturated fatty acid
ORAC	Oxygen radical absorption capacity
SDS-PAGE	Sodium dodecyl sulfate-polyacrylamide gel electrophoresis
SFA	Saturated fatty acid
SPE	Solid phase extraction
SQMG	Sulfoquinovosyl monoacylglycerol
SQDG	Sulfoquinovosyl diacylglycerol
PC	Phosphatidylcholine
PI	Protein isolate
PUFA	Polyunsaturated fatty acid
TAG	Triacylglycerol
TE	Trolox equivalents
TIC	Total ion chromatogram
UHPLC	Ultra-high performance liquid chromatography
