# Supplementary material for: Biochemical Characterization of Lipids, Proteins, and Polysaccharides from the Marine Diatom Phaeodactylum tricornutum Cultivated in Pilot-Scale Photobioreactors
Source: Molecules. 2026 Mar 18;31(6):1017. doi: 10.3390/molecules31061017 (PMC13029753; doi:10.3390/molecules31061017)
Supplement: Supplementary file 1 [file molecules-31-01017-s001.zip › molecules-4169268-supplementary.pdf]

SUPPLEMENTRY DOCUMENTS

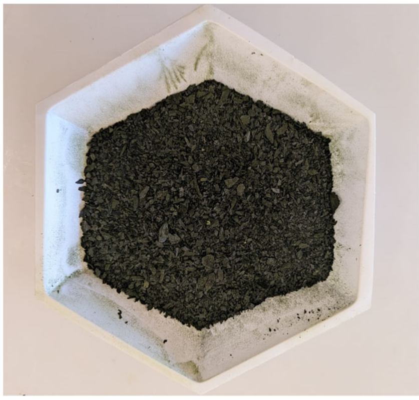

Figure S1. Freeze-dried biomass of *P. tricornutum*.

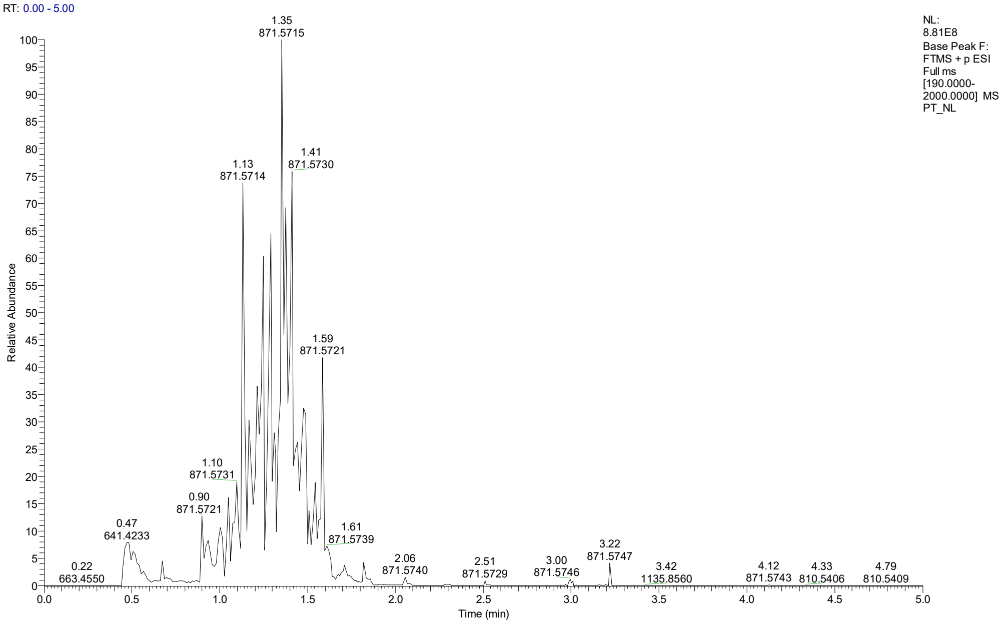

Figure S2. Total ion chromatogram (TIC) of neutral lipid fraction in Electrospray ionization (ESI) positive mode.

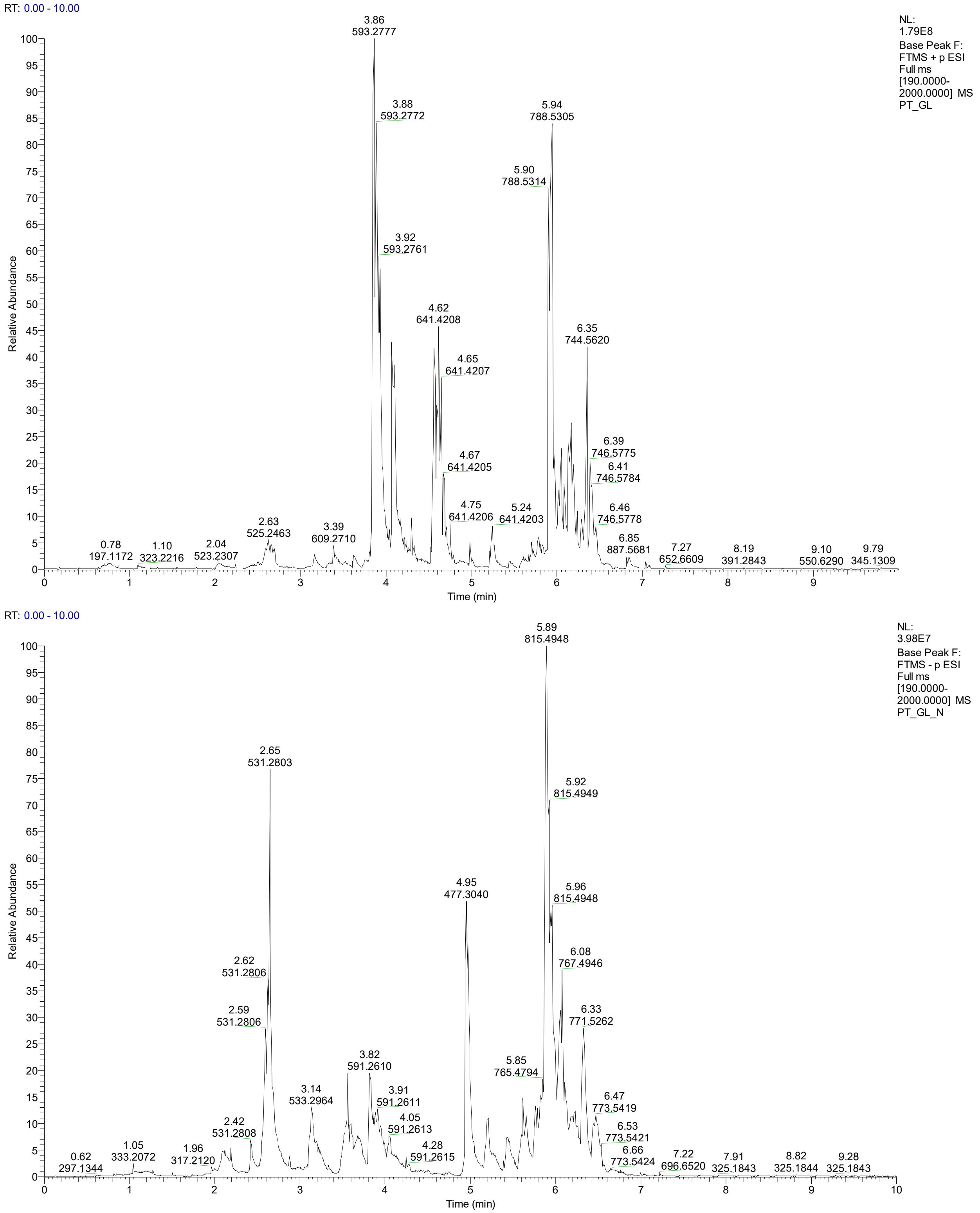

Figure S3. TIC of glycolipid lipid fraction in ESI positive (top) and negative mode (bottom).

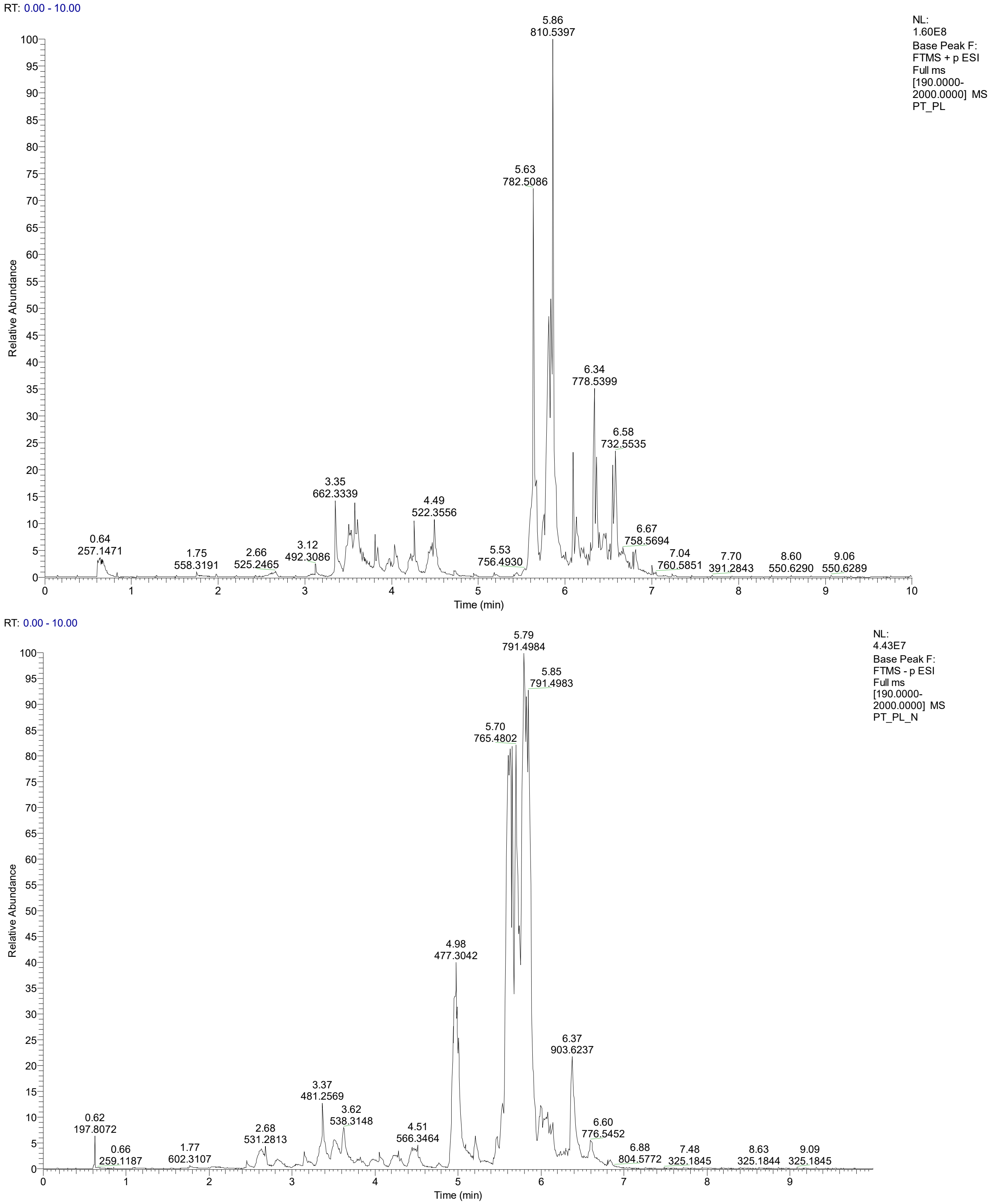

Figure S4. TIC of phospholipid fraction in ESI positive (top) and negative mode (bottom).

Table S1. Triacylglycerols (TAGs) identified in neutral lipid fraction by Ultra high performance liquid chromatography-high resolution mass spectrometry (UHPLC-HRMS) analysis. $[M + NH_4]^+$ ion was used for the database search.

RT (min)	Exact Mass (m/z)	Observed Mass (m/z)	Error (ppm)	TAG C:DB	Relative Abundance (%)
1.48	914.7232	914.7233	-0.11	TAG 56:11	2.6
1.59	864.7076	864.7059	1.97	TAG 52:8	1.9
1.71	892.7389	892.7412	-2.58	TAG 54:8	9.7
1.82	840.7076	840.7101	-2.97	TAG 50:6	1.2
1.82	866.7232	866.7250	-2.08	TAG 52:7	3.6
2.04	816.7076	816.7097	-2.57	TAG 48:4	1.5
2.04	842.7232	842.7256	-2.85	TAG 50:5	1.0
2.04	868.7389	868.7405	-1.84	TAG 52:6	3.2
2.04	894.7545	894.7558	-1.45	TAG 54:7	13.7
2.28	792.7076	792.7089	-1.64	TAG 46:2	1.2
2.28	818.7232	818.7245	-1.59	TAG 48:3	3.4
2.28	844.7389	844.7411	-2.60	TAG 50:4	1.2
2.28	870.7545	870.7564	-2.18	TAG 52:5	4.4
2.30	896.7702	896.7709	-0.78	TAG 54:6	14.1
2.67	794.7232	794.7245	-1.64	TAG 46:1	1.5
2.73	820.7389	820.7399	-1.22	TAG 48:2	5.8
2.73	846.7545	846.7562	-2.01	TAG 50:3	2.6
2.73	872.7702	872.7702	0.00	TAG 52:4	5.0
2.73	898.7858	898.7876	-2.00	TAG 54:5	5.9
2.73	924.8015	924.8018	-0.32	TAG 56:6	0.4
3.08	822.7545	822.7554	-1.09	TAG 48:1	3.2
3.08	874.7858	874.7858	0.00	TAG 52:3	1.9
3.10	848.7702	848.7709	-0.82	TAG 50:2	2.8
3.11	900.8015	900.8031	-1.78	TAG 54:4	3.2
3.22	926.8171	926.8140	3.34	TAG 56:5	0.7
3.45	850.7858	850.7868	-1.18	TAG 50:1	0.8
3.45	876.8015	876.8032	-1.94	TAG 52:2	0.9
3.45	928.8328	928.8337	-0.97	TAG 56:4	0.7
3.53	902.8171	902.8179	-0.89	TAG 54:3	1.1
3.54	954.8481	954.8510	-3.04	TAG 58:3	0.1
3.88	904.8328	904.8338	-1.11	TAG 54:2	0.3
3.88	930.8484	930.8527	-4.62	TAG 56:3	0.3
3.90	956.8641	956.8647	-0.63	TAG 58:4	0.2
3.91	982.8797	982.8798	-0.10	TAG 60:5	0.0
4.33	932.8641	932.8647	-0.64	TAG 56:2	0.1

Low abundance High abundance

Table S2. Monogalactosylmonoacylglycerol (MGMG), monogalactosyldiacylglycerol (MGDG), digalactosyldiacylglycerol (DGDG) and sulfoquinovosyl monoacylglycerol (SQMG) and sulfoquinovosyl diacylglycerol (SGDG) identified in phospholipid fraction and their relative intensities.

RT (min)	Exact Mass (m/z)	Observed Mass (m/z)	Error (ppm)	Glycolipid	RI (%)	R1/R2
**MGMG, MDDG and DGDG (M + NH_4)$^+$**						
3.19	506.3329	506.3326	0.6	MGMG 16:2	0.5	16:02*
5.90	902.5835	902.5844	-1	DGDG 32:4	1.0	NI
5.91	952.5992	952.5994	-0.2	DGDG 36:7	25.2	16:2/20:5*
5.92	788.5307	788.5306	0.1	MGDG 36:8	1.9	16:3/20:5*
5.94	764.5307	764.5321	-1.8	MGDG 34:6	0.3	16:2/16:4*
6.00	928.5992	928.6002	-1.1	DGDG 34:5	0.3	NI
6.05	740.5307	740.5309	-0.3	MGDG 32:4	0.8	16:1/16:3; 16:2/16:2
6.05	954.6148	954.6144	0.4	DGDG 36:6	37.4	16:1/20:5*
6.09	904.5992	904.6000	-0.9	DGDG 32:3	1.8	16:1/16:2*
6.13	742.5464	742.5463	0.1	MGDG 32:3	0.3	16:1/16:2; 16:0/16:3*
6.13	906.6148	906.6151	-0.3	DGDG 32:2	14.0	16:1/16:1
6.17	930.6148	930.6132	1.7	DGDG 34:4	0.3	NI
6.33	956.6305	956.6312	-0.7	DGDG 36:5	8.0	NI
6.35	744.5620	744.5618	0.3	MGDG 32:2	0.3	16:1/16:1
6.35	908.6305	908.6302	0.3	DGDG 32:1	6.5	16:0/16:1*
6.35	934.6461	934.6461	0	DGDG 34:2	1.2	NI
6.39	746.5777	746.5776	0.1	MGDG 32:1	0.4	16:1/16:0
**SQMG and SQDG (M – H)$^-$**						
2.45	527.2532	527.2535	-0.6	SQMG 14:0	0.3	14:0*
2.82	553.2688	553.2690	-0.4	SQMG 16:1	0.7	16:1*
3.39	555.2845	555.2845	0.0	SQMG 16:0	1.4	16:0*
5.46	761.4515	761.4519	-0.5	SQDG 30:2	0.8	14:0/16:2*
5.53	737.4515	737.4521	-0.8	SQDG 28:0	4.0	14:0/14:0
5.53	811.4672	811.4679	-0.9	SQDG 34:5	1.2	20:5/14:0
5.58	787.4672	787.4675	-0.4	SQDG 32:3	0.4	16:1/16:2*
5.62	837.4828	837.4830	-0.2	SQDG 36:6	0.5	16:1/20:5*
5.65	763.4672	763.4668	0.5	SQDG 30:1	23.9	10:0/16:1 (major)
5.68	789.4828	789.4828	0.0	SQDG 32:2	4.6	16:0/16:2 (major); 16:1/16:1
5.70	839.4985	839.4981	0.4	SQDG 36:5	6.4	16:0/20:5
5.70	765.4828	765.4802	3.4	SQDG 30:0	17.2	14:0/16:0
5.85	791.4985	791.4983	0.2	SQDG 32:1	32.8	16:1/16:0

5.90	817.5141	817.5145	-0.5	SQDG 34:2	1.1	16:1/18:1; 18:2/16:0
6.00	819.5298	819.5300	-0.2	SQDG 34.1	1.1	16:0/18:1; 16:1/18:0
6.37	903.6237	903.6237	0.0	SQDG 40:1	3.9	16:1/24:0

* position of fatty acid acyl chain is not assigned, NI – fatty acid acyl chain is not defined because of lack of diogenstic fragment ions, Low abundance High abundance

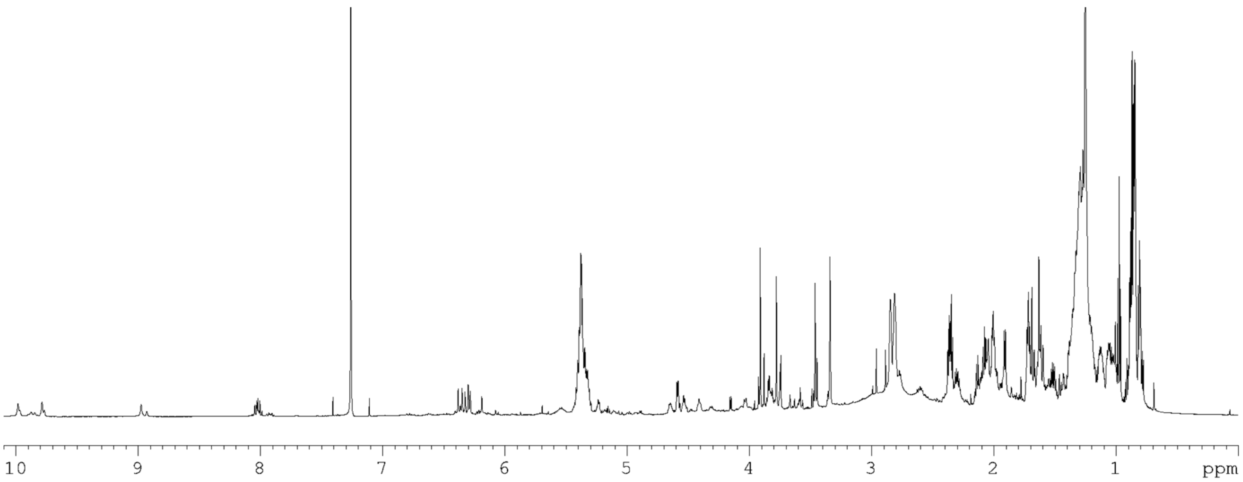

Figure S5. ^{1}H NMR spectrum of neutral lipid fraction recorded in 700 MHz NMR Spectrometer.

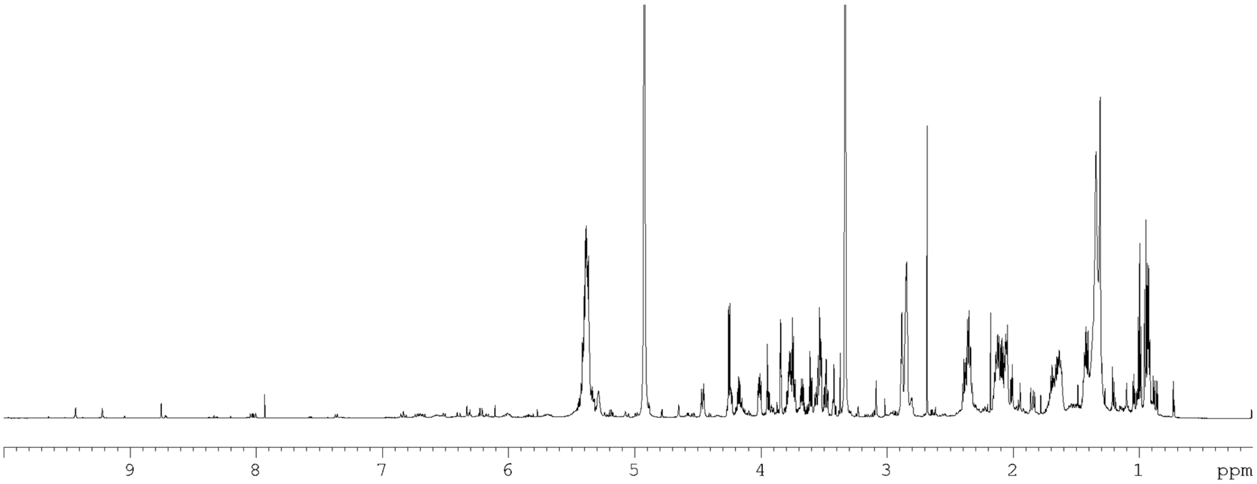

Figure S6. ^{1}H NMR spectrum of glycolipid fraction recorded in 700 MHz NMR Spectrometer.

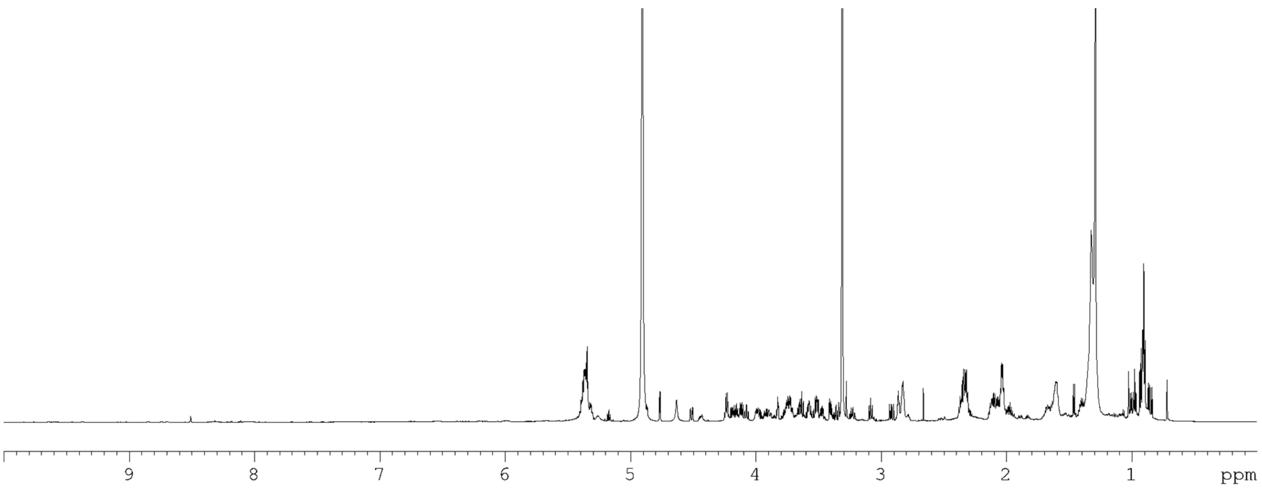

Figure S7. ^{1}H NMR spectrum of phospholipid fraction recorded in 700 MHz NMR Spectrometer.

Combi Flash chromatography purification of phospholipid fraction: The phospholipid fraction 500 mg was subjected for Combi Flash chromatography (Teledyne Isco, Linciln, NE) using silica gel cartage 40 g eluting with dichloromethane methanol gradient with flow rate of 60 mL/min approximately 20 mL/fraction. Fraction description are follows:

Table S3. Combi Flash chromatography pooled fractions, along with their corresponding weight and percentage (%) recoveries.

**Fraction**	**Weight (mg)**	**% Recovery**	**Comments based on ^{1}H NMR spectrum**
1-20	-	-	No compounds discarded
21-24	17.6	3.5	Pigment mainly chlorophyll related products
25-27	56.0	11.2	MGDG
28-33	66.0	13.2	mixture
34-36	84.6	16.9	DGDG
37-40	175.0	35.0	SQDG
41-55	100.2	20.0	mixture including nonlipid metabolites
56-60	4.4	0.9	PC
**Total % Recovery**	503.7	100.7	

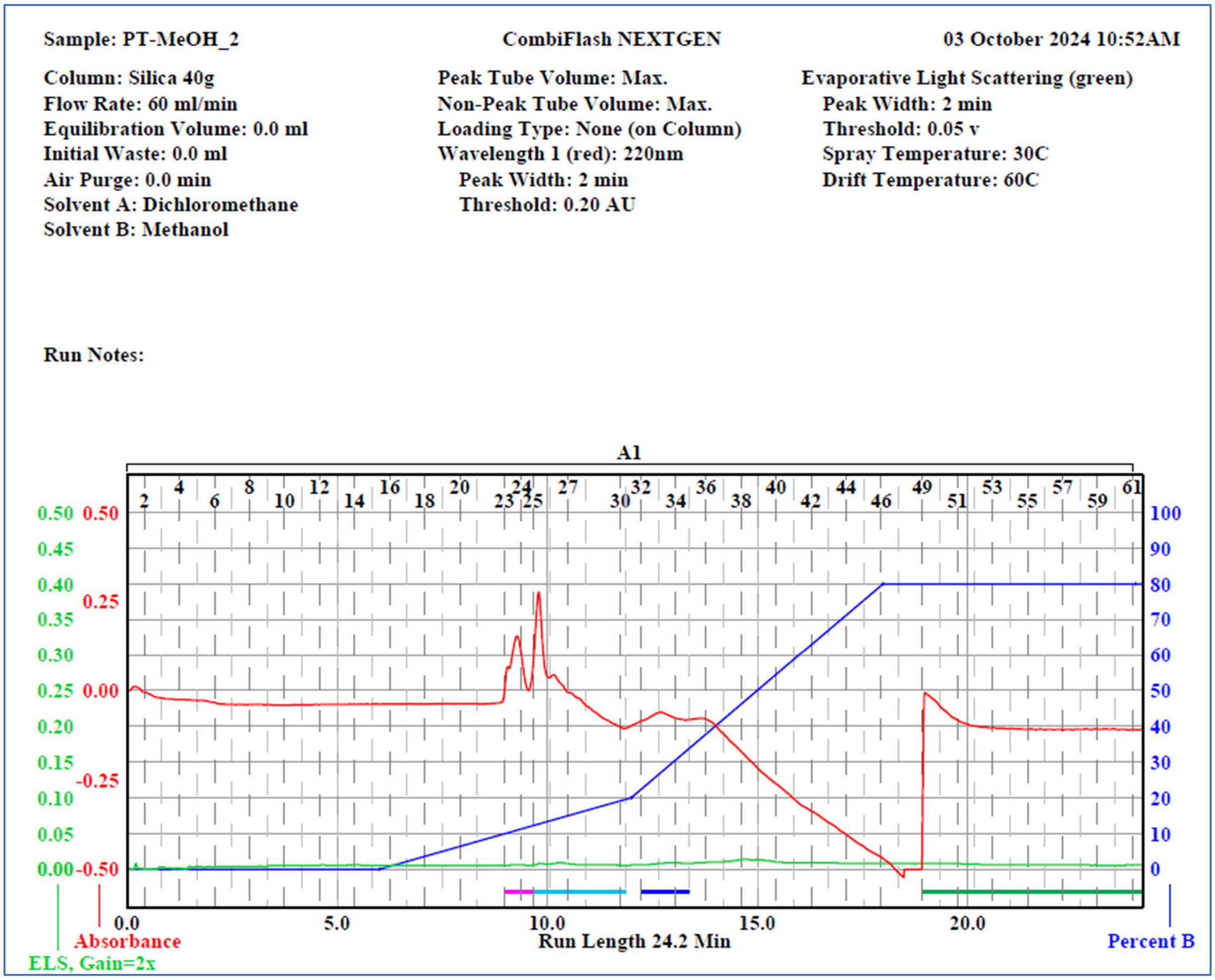

Figure S8. Combi Flash Chromatography chromatogram of phospholipid fraction.

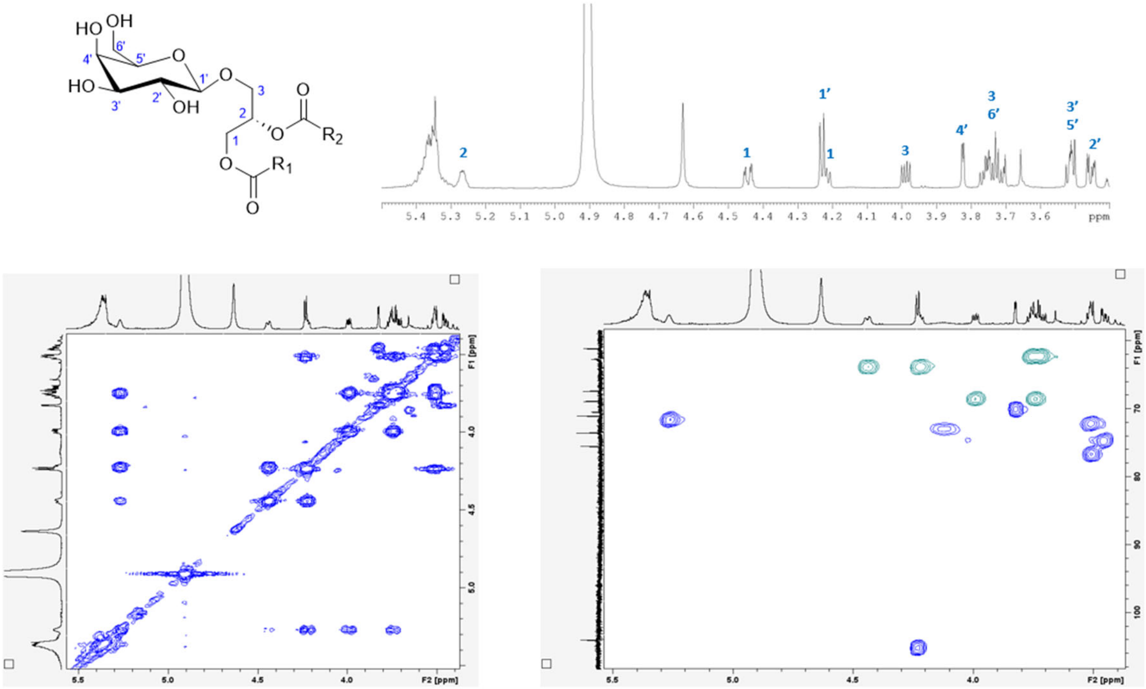

Figure S9. NMR spectrum of monogalactosyldiacylglycerol (MGDG) rich fraction clockwise from top left – representative chemical structure of MGDG where R_1 and R_2 are fatty acid acyl chain, part of the ^{1}H NMR spectrum between 3.4 ppm – 5.50 ppm having key diogenitic proton NMR signals belong to sugar and glycerol moieties, part of heteronuclear single quantum coherence (HSQC) and part of correlation spectroscopy (COSY spectrum).

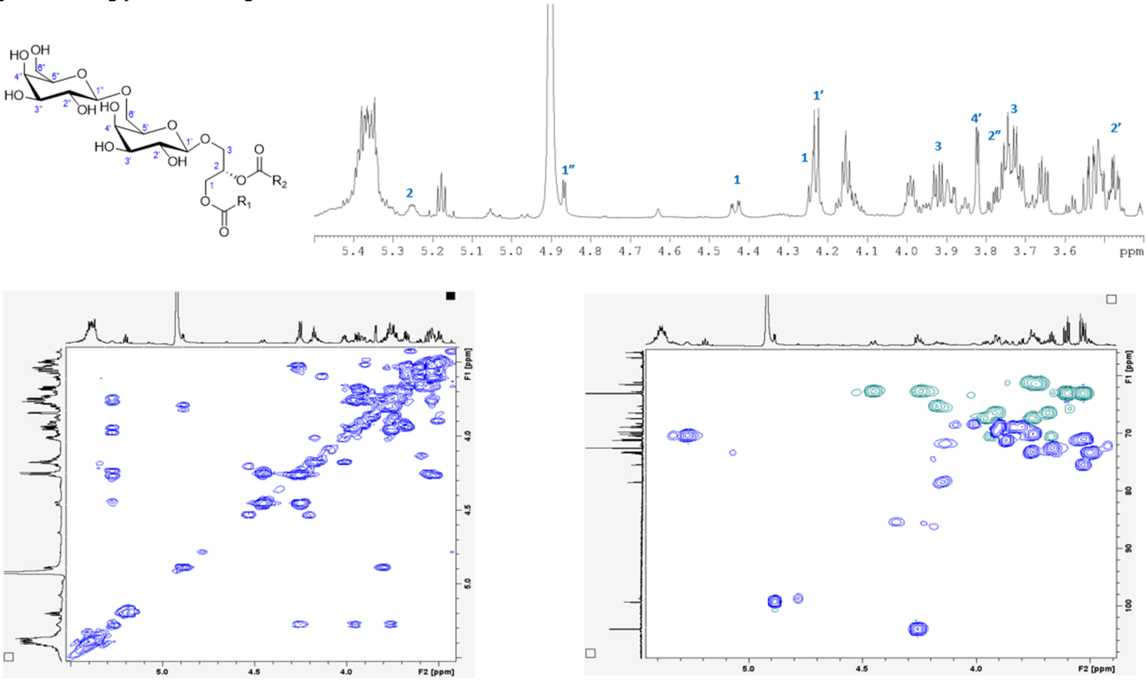

Figure S10. NMR spectrum of digalactosyldiacylglycerol (DGDG) rich fraction clockwise from top left – representative chemical structure of DGDG where R_1 and R_2 are fatty acid acyl chain, part of the ^{1}H NMR

spectrum between 3.4 ppm – 5.50 ppm having key diogenitic proton NMR signals belong to sugar and glycerol moieties, part of HSQC and part of COSY spectrum.

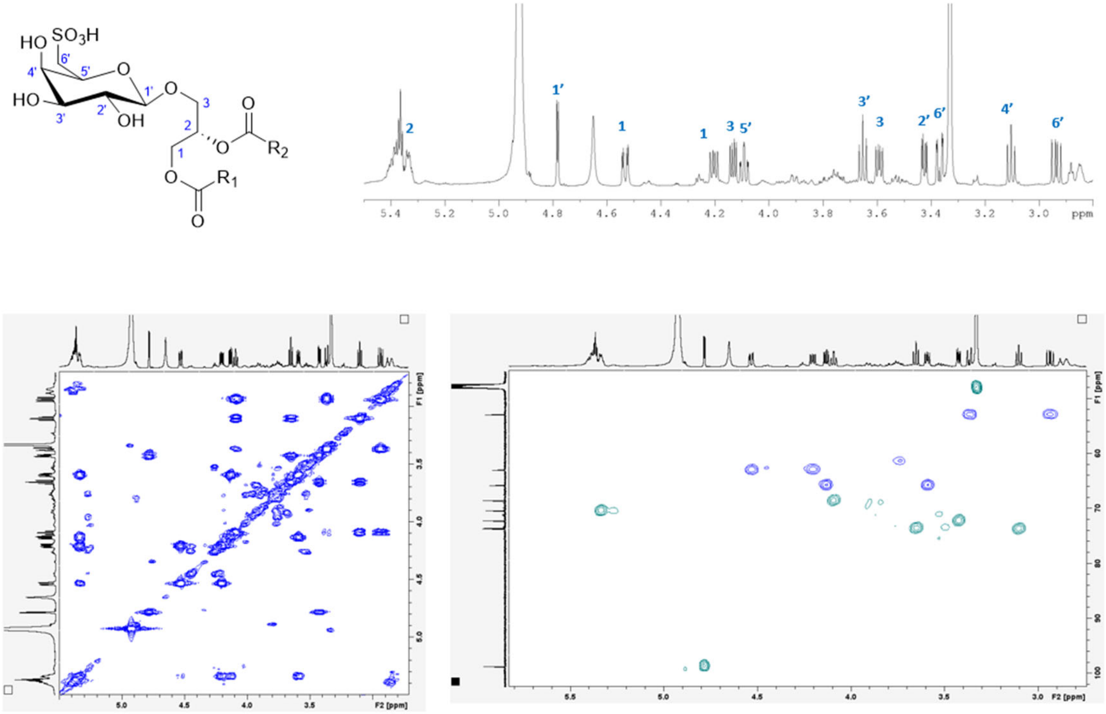

Figure S11. NMR spectrum of sulfoquinovosyl diacylglycerol (SQDG) rich fraction clockwise from top left – representative chemical structure of SQDG where R_1 and R_2 are fatty acid acyl chain, part of the ^1H NMR spectrum between 3.4 ppm – 5.50 ppm having key diogenitic proton NMR signals belong to sugar and glycerol moieties, part of HSQC and part of COSY spectrum.

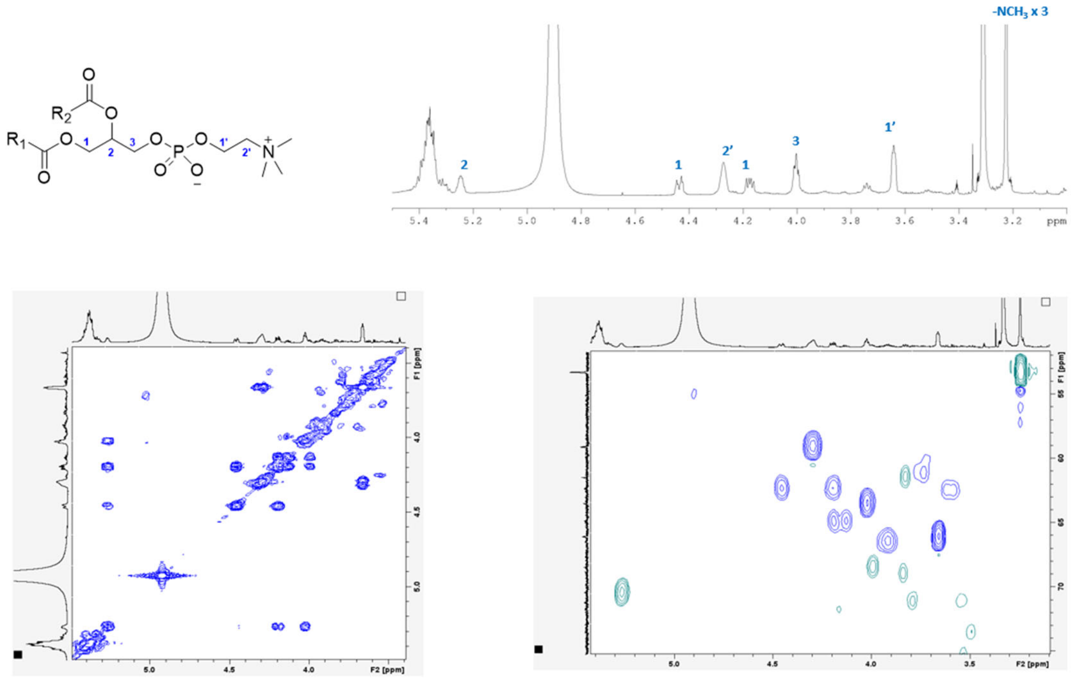

Figure S12. NMR spectrum of phosphatidylcholine (PC) rich fraction clockwise from top left – representative chemical structure of PC where R1 and R2 are fatty acid acyl chain, part of the ^{1}H NMR spectrum between 3.4 ppm – 5.50 ppm having key diogenitic proton NMR signals belong to sugar and glycerol moieties, part of HSQC and part of COSY spectrum

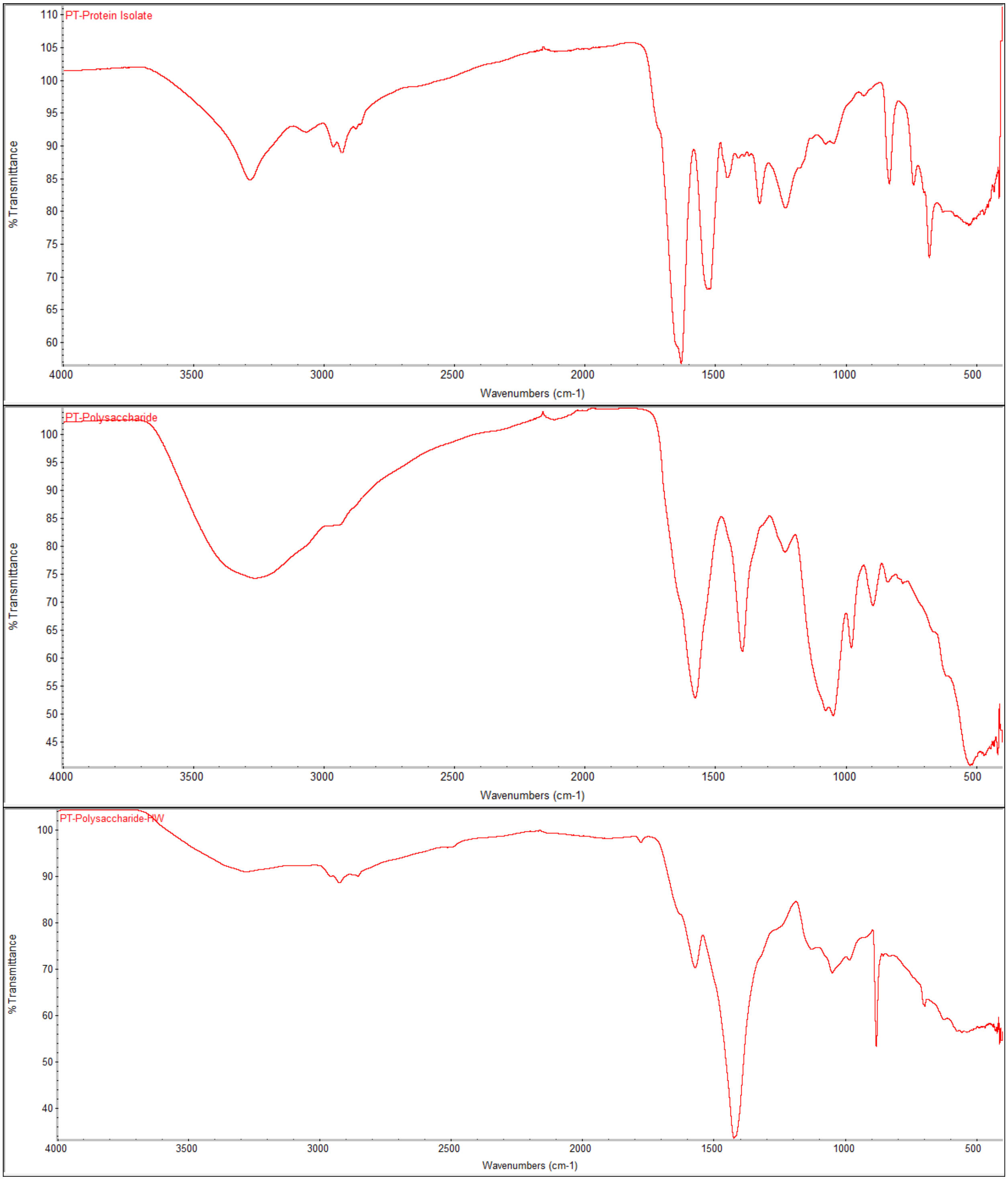

Figure S13. FT-IR spectrum of protein isolate (top) and polysaccharide extracted by alkaline extraction followed by EtOH precipitation (middle) and polysaccharide derived from hot water extraction of the residual biomass after alkaline extraction of *P. tricornutum* (bottom)

SDS-PAGE gel electrophoresis: Protein isolate (PI) extracted from *P. tricornutum* were dissolved in lysis buffer at 2.0, 4.0 and 8.0 mg/mL containing 50 mM Tris-HCl at pH 7.4, 150 mM NaCl, 1% Triton x-100 (v/v) and 0.1% SDS (w/v). In a separate experiment protein were directly extracted with lysis buffer sonicating for 5 min at room temperature at 5.0, 10.0 and 20.0 mg/mL concentration. The resulting mixture were centrifuged at 13000 rpm for 5 minutes. The supernatant derived from direct extraction or PI solution (30 ◎L) were mixed with 4x laemmli buffer (10 ◎L, Bio-Rad, Hercules, CA, USA) with and without reducing agent dithiothreitol 2% (w/v) and incubated at 90°C for 5 minutes in a water bath. Samples (20 ◎L) were then deposited in the wells of an SDS-PAGE gel (12% acrylamide) and run in 10x Tris/Glycine/SDS buffer (Bio-Rad, Hercules, CA, USA) at 150 v until the tracking dye reached the bottom of the gel. The gel was dyed and de-stained using Bio-Safe Coomassie G-250 stain (Bio-Rad, Hercules, CA, USA) according to manufacturer's instruction. The final image of the gel was recorded using a Molecular Imager ChemiDOC XRS with Image Lab Software (Bio-Rad, Hercules, CA, USA).

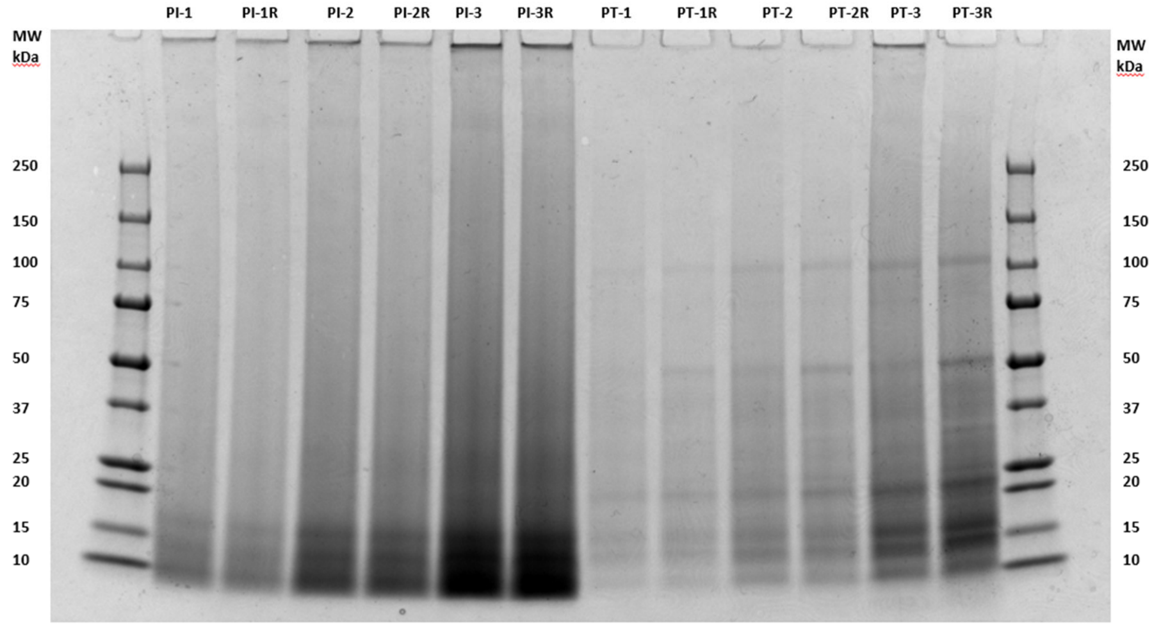

Figure S14. SDS-PAGE profiles of protein isolate extracted from *P. tricornaturm* (lane 2-7), protein directly extracted from freeze-fry biomass using lysis buffer (lanes 8-13) and protein standard (lanes 1 and 14).

PI-I: 2 mg/mL, PI-1R: 2 mg/mL with reducing agent, PI-2: 4 mg/mL, PI-2R: 4 mg/mL with reducing agent, PI-3: 8 mg/mL, PI-3R: 8 mg/mL with reducing agent, PT-I: 5 mg/mL, PT-I: 5 mg/mL with reducing agent, PT-2: 10 mg/mL, PT-2R: 10 mg/mL with reducing agent, PT-3: 20 mg/mL and PT-3R: 20 mg/mL with reducing agent.

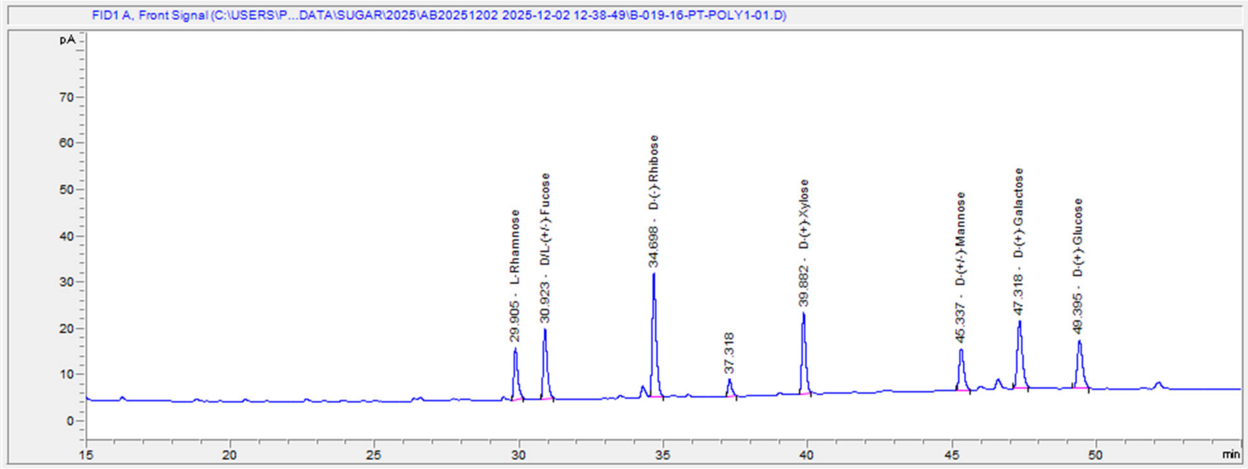

Monomer	RT (min)	RI (%)	Monomer	RT (min)	RI (%)
Rhamnose	29.90	8.8	Mannose	45.36	8.7
Fucose	30.92	12.3	Unknown-4	46.00	0.6
Unknown-1	34.31	1.9	Unknown-5	46.60	1.9
Rhibose	34.68	21.4	Galactose	47.34	15.2
Unknown-2	37.31	2.8	Glucose	49.39	11.4
Xylose	39.87	13.5	Myo-inositol	52.01	1.5
Unknown-3	43.13	0.0			

Figure S15. Monomer analysis of polysaccharide extracted by EtOH precipitation. GC chromatogram (top) and relative percentage of the monomer detected (bottom).

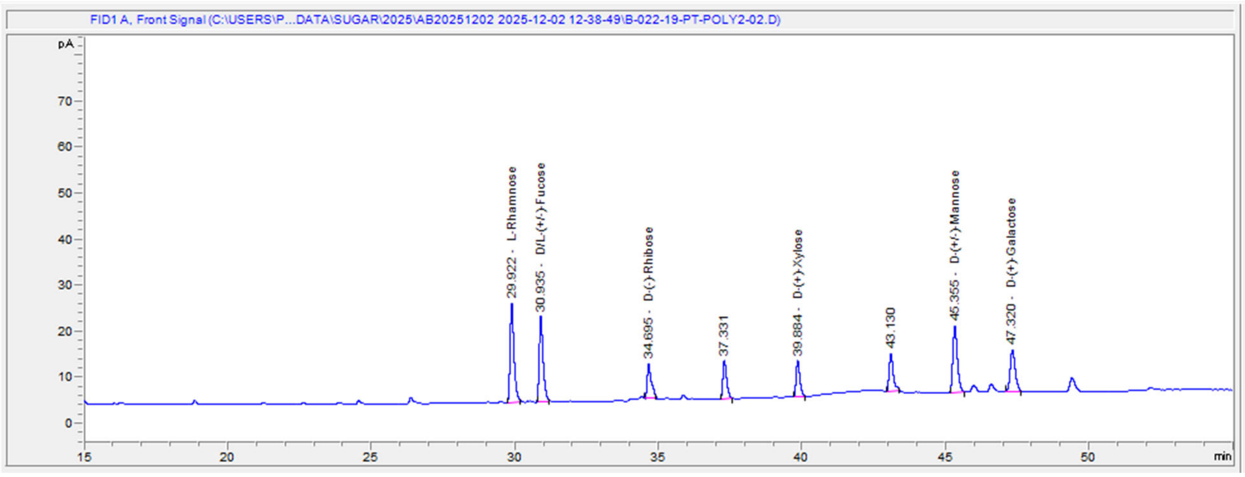

Monomer	Retention Time (min)	Relative Intensity (%)	Monomer	Retention Time (min)	Relative Intensity (%)
Rhamnose	29.90	19.9	Mannose	45.36	15.7

Fucose	30.92	17.0	Unknown-4	46.00	1.2
Unknown-1	34.31	0.0	Unknown-5	46.60	1.7
Rhibose	34.68	7.3	Galactose	47.34	11.2
Unknown-2	37.31	7.6	Glucose	49.39	2.2
Xylose	39.87	7.1	Myo-inositol	52.01	0.0
Unknown-3	43.13	9.2			

Figure S16. Monomer analysis of polysaccharide extracted by hot water. GC chromatogram (top) and relative percentage of the monomer detected (bottom).

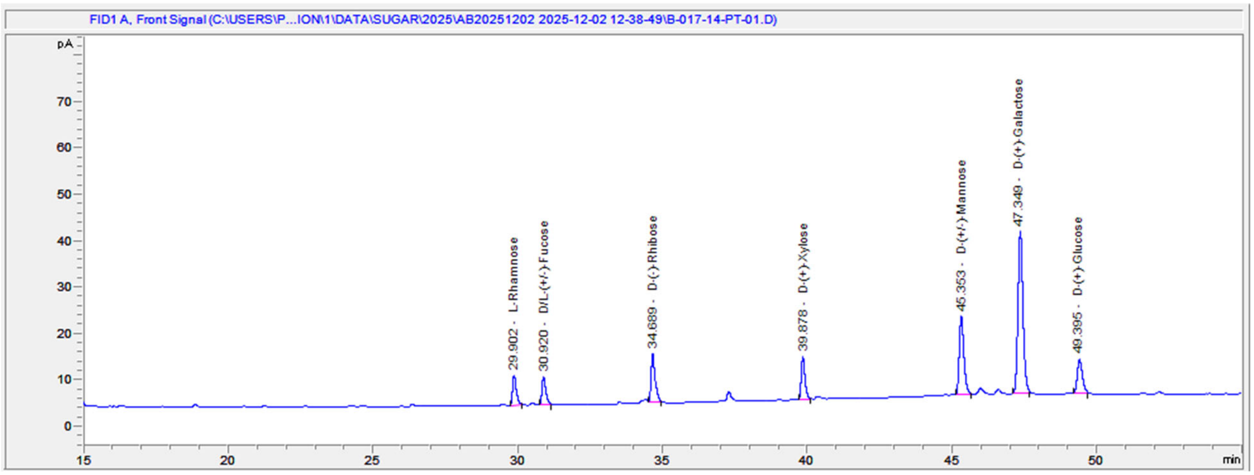

Monomer	RT (min)	RI (%)	Monomer	RT (min)	RI (%)
Rhamnose	29.90	5.6	Mannose	45.36	17.9
Fucose	30.92	5.4	Unknown-4	46.00	1.0
Unknown-1	34.31	0.0	Unknown-5	46.60	0.9
Rhibose	34.68	9.5	Galactose	47.34	40.7
Unknown-2	37.31	1.8	Glucose	49.39	8.8
Xylose	39.87	7.9	Myo-inositol	52.01	0.4
Unknown-3	43.13	0.0			

Figure S17. Monomer analysis of *P. tricornutum* whole biomass. Gas chromatography (GC) chromatogram (top) and relative percentage of the monomer detected (bottom).

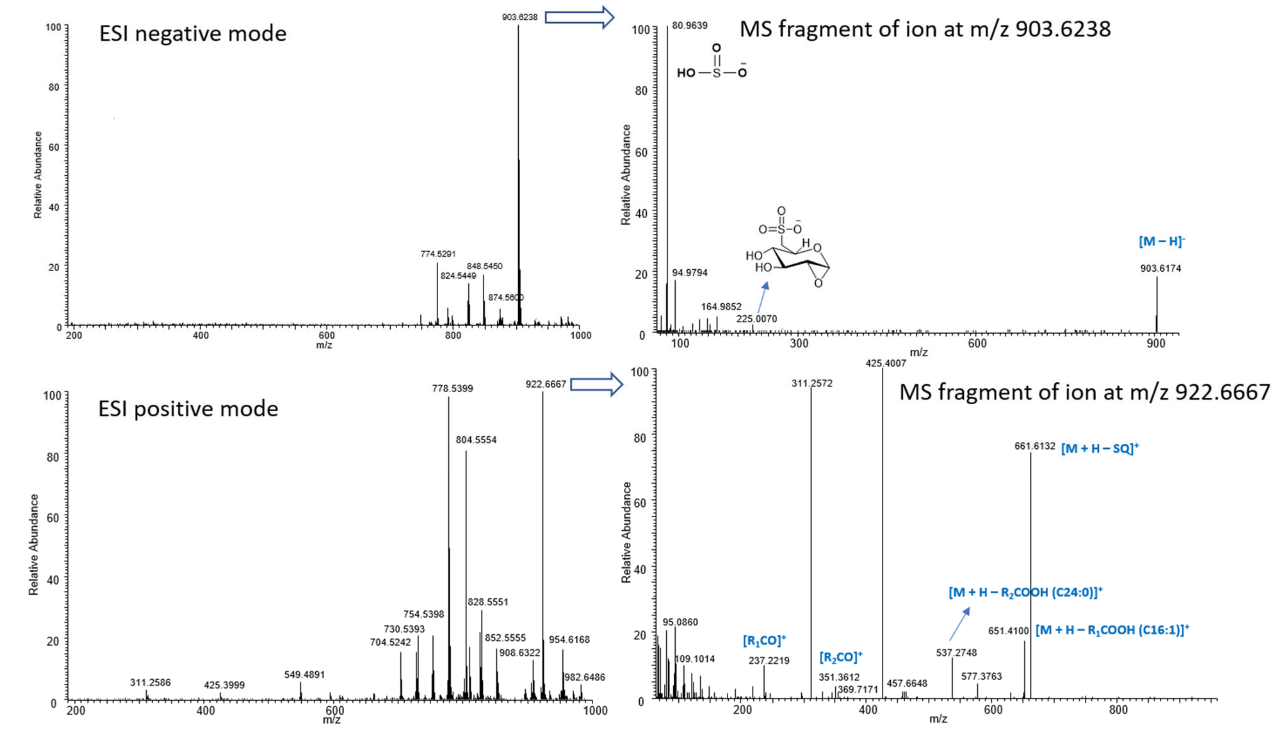

Figure S18. MS spectra of SQDG (*sn*-1:C16:1 / *sn*-2:C24:0) eluted at 6.37 min with fragmentation in positive and negative mode.

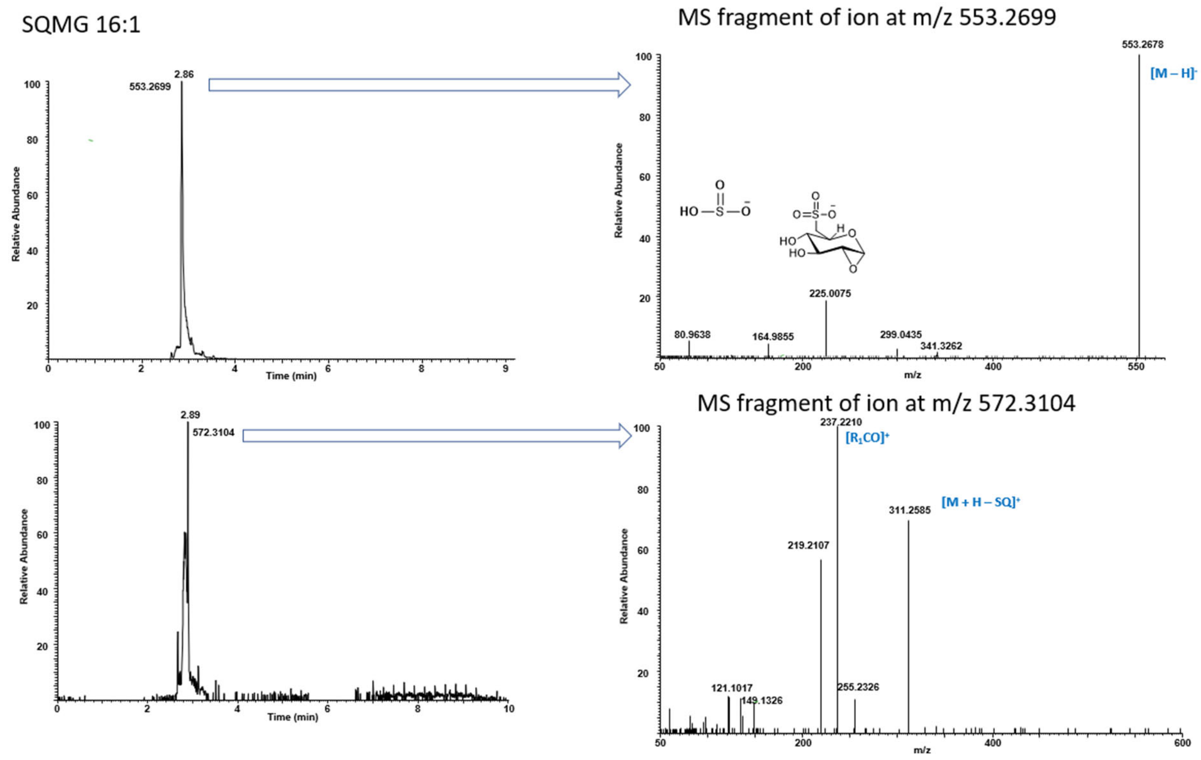

Figure S19. Total extracted ion of sulfoquinovosyl monoacylglycerol (SQMG) - SQMG (C16:1) eluted at 6.37 min and MSMS fragmentation in negative mode (top rows) and positive mode (bottom rows).

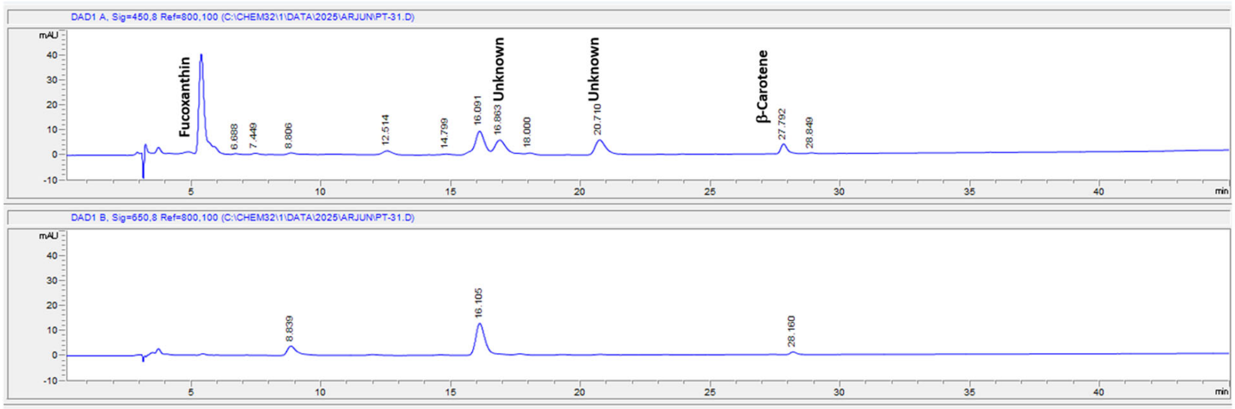

Figure S20. High performance liquid chromatography (HPLC) chromatogram of $CHCl_3$/MeOH (1:1) extract of *P. tricornutum* at 450 nm (top) and 665 nm (bottom).
